# The Diverse Functional Roles of Elongation Factor Tu (EF-Tu) in Microbial Pathogenesis

**DOI:** 10.3389/fmicb.2019.02351

**Published:** 2019-10-24

**Authors:** Kate L. Harvey, Veronica M. Jarocki, Ian G. Charles, Steven P. Djordjevic

**Affiliations:** ^1^The ithree Institute, University of Technology Sydney, Ultimo, NSW, Australia; ^2^Quadram Institute, Norwich, United Kingdom; ^3^Norwich Medical School, Norwich, United Kingdom

**Keywords:** EF-Tu, moonlighting, bacteria, adhesion, chaperone activities

## Abstract

Elongation factor thermal unstable Tu (EF-Tu) is a G protein that catalyzes the binding of aminoacyl-tRNA to the A-site of the ribosome inside living cells. Structural and biochemical studies have described the complex interactions needed to effect canonical function. However, EF-Tu has evolved the capacity to execute diverse functions on the extracellular surface of both eukaryote and prokaryote cells. EF-Tu can traffic to, and is retained on, cell surfaces where can interact with membrane receptors and with extracellular matrix on the surface of plant and animal cells. Our structural studies indicate that short linear motifs (SLiMs) in surface exposed, non-conserved regions of the molecule may play a key role in the moonlighting functions ascribed to this ancient, highly abundant protein. Here we explore the diverse moonlighting functions relating to pathogenesis of EF-Tu in bacteria and examine putative SLiMs on surface-exposed regions of the molecule.

## Introduction

Elongation Factor Thermo Unstable (EF-Tu) is one the most abundant proteins found in bacteria, comprising up to 6% of the total protein expressed in *Escherichia coli* (Furano, 1975) and as high as 10% of the total protein expressed in the genome reduced pathogen *Mycoplasma pneumoniae* (Dallo et al., 2002). The primary, canonical function of EF-Tu is to transport aminoacylated tRNAs to the ribosome (Sprinzl, 1994). Ef-Tu has been a therapeutic target for antibiotics (elfamycins) since the 1970s (Wolf et al., 1974; Prezioso et al., 2017). However, current issues with elfamycins’ poor pharmacokinetics and solubility has prevented their commercialization as therapeutic agents (Prezioso et al., 2017).

Diverse functions have been ascribed to EF-Tu many of which include important virulence traits in Gram positive and Gram-negative pathogenic bacteria. To effect alternate virulence-associated functions, including adhesion to host extracellular matrix components, EF-Tu must gain access and be retained on the extracellular surface. This poses a challenge as signal secretion motifs are absent in this highly structured protein, and motifs required for binding diverse host cell surface receptor and matrix molecules must evolve without jeopardizing structural constraints needed to execute canonical function as a G protein. Here we refer to secondary functions as “moonlighting” functions. The concept of protein moonlighting is well established in eukaryotes (Jeffery, 1999; Huberts and van der Klei, 2010; Petit et al., 2014; Min et al., 2016; Yoon et al., 2018), and is rapidly gaining traction in prokaryotes (Henderson and Martin, 2011, 2013; Wang et al., 2013b; Kainulainen and Korhonen, 2014; Jeffery, 2018; Ebner and Götz, 2019) indicating that it is an ancient and evolutionally conserved phenomenon. Although EF-Tu executes various functions in eukaryotes, a review of the moonlighting roles of EF-Tu in bacteria is lacking. Therefore, this review has a focus to discuss the ever-expanding moonlighting roles of EF-Tu in prokaryotes, and how these roles relate to pathogenesis.

## Structure and Function of EF-Tu

### Structural Analysis of EF-Tu

**FIGURE 1:**
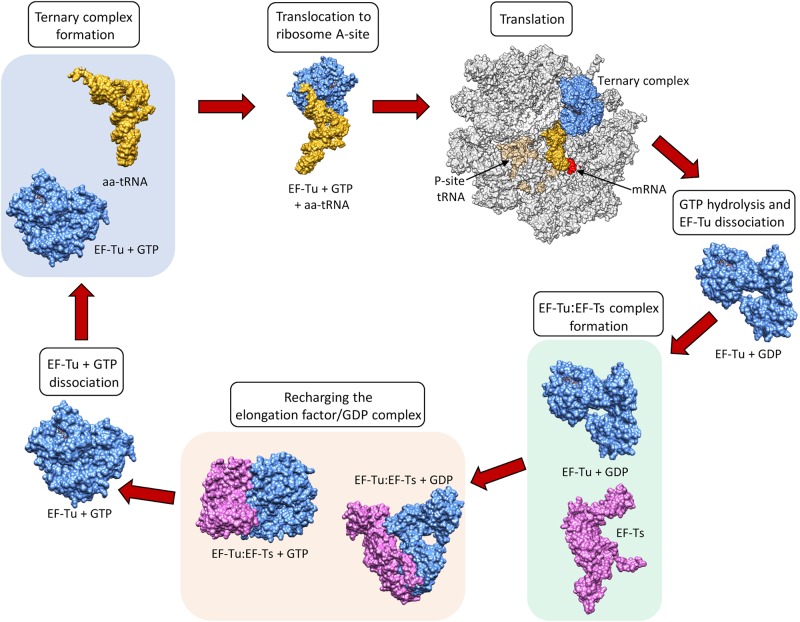
The canonical role of EF-Tu in translation. Structures sourced from protein databank (PDB), accession numbers 1DG1, 5OPD, 1B23, 4PC7, 1EFT, 1EFT. EF-Ts monomer structure obtained using Phyre2.

Elongation factors (Table 1) in bacteria (e.g., EF-Tu also known as EF1A) and in eukaryotes (e.g., the eukaryotic Elongation Factor 1 Complex [eEF1A]) all have the same primary and critical function to shuttle aminoacylated tRNAs to the ribosome during protein translation. A codon–anticodon system ensures that the correct amino acid is added to the growing protein chain, a process that consumes guanosine triphosphate (GTP) prior to releasing the elongation factor from the aminoacyl tRNA. However, bacteria and eukaryotes differ in the mechanism by which they recharge the elongation factor/guanosine diphosphate (GDP) complex. This recharging function is executed by the Elongation Factor Thermo stable (EF-Ts) in prokaryotes and by eukaryotic Elongation Factor 1B (eEF1B) in eukaryotes (Cacan et al., 2013) (Figure 1).

**TABLE 1:** Elongation Factors in eukaryotes and their equivalent title in prokaryotes.

**Eukaryotic protein**	**Prokaryotic equivalent**
eEF1/EF-1	EF1/EF-T
eEF1A/(e)EF-1α	EF1A/EF-Tu
$\begin{array}{l} \text{eEF1A1/eEF1α1}\\ \text{eEF1A2/eEF1α2} \end{array}\}\;\;2\;\text{isoforms}$	
eEF1B/(e)EF-1β	EF1B/EF-Ts
eEF1Bβγδ (animals) 3 subunits	
eEF1Bαβγ (yeast) 2 subunits	
eEF1Bβγδ (plants) 3 subunits	
eEF2/EF-2	EF2/EF-G
eEF3/EF-3	N/A

*Both the new nomenclature and the old are shown below in the format of current/old. Although new nomenclature has been established for over a decade, many articles still use the old nomenclature. Indeed, the old nomenclature is more widely used in the prokaryotic literature and that is why EF1A is referred to as EF-Tu in this review. Adapted from Ejiri (2002) and Sasikumar et al. (2012).*

EF-Tu is comprised of three domains known as domains i, ii and iii which have evolved a high degree of molecular flexibility. To perform its canonical function, EF-Tu must form a functional binding pocket for an aminoacyl-tRNA, and to achieve this, domain i must become aligned more closely to domains ii and iii (i.e., they must move by around 90°) (Kjeldgaard et al., 1993). The extent of intramolecular movement needed to accommodate the aminoacyl-tRNA is about one third of the protein’s total diameter, indicating how significant this conformational change is (Sprinzl, 1994). Once the incoming aminoacyl-tRNA has docked with the mRNA, GTPase activity induces a reverse conformational change enabling the release of EF-Tu from the ribosome (Polekhina et al., 1996). The structural and functional constraints needed to execute these critical molecular interactions ensure that key domains within EF-Tu evolve slowly compared to molecules that perform their functions on the cell surface, where they face constant challenge from the host’s immunological defenses and undergo diversifying selection. As such, EF-Tu is considered to be an ancient molecule that is comprised of domains that are highly conserved in phylogenetically diverse prokaryotes (Filer and Furano, 1980). This sequence conservation extends to EF-Tu homologs in eukaryotes, which have also evolved a similar overall protein synthesis pathway (Ejiri, 2002).

### Genetic Evolution of EF-Tu

In bacteria, EF-Tu is encoded by the *tuf* gene. *tuf* has a highly conserved genomic location and amino acid sequence, and has been used in the construction of phylogenetic trees for species discrimination (Iwabe et al., 1989; Baldauf et al., 1996; Mignard and Flandrois, 2007; Shin et al., 2009; Li et al., 2012; Caamano-Antelo et al., 2015). Amongst different bacterial species the EF-Tu sequences have less than 30% sequence divergence (Lathe and Bork, 2001). Low G + C Gram positive bacteria carry only a single copy of *tuf* (Ke et al., 2000). In contrast, many enteric bacteria have two copies (*tufA* and *tufB*) while three *tuf*-like genes have been identified in *Streptomyces ramocissimus* (Filer and Furano, 1981; Vijgenboom et al., 1994). In species with two copies of the gene, the two genes differ by less than 1.4%, based on nucleotide comparison (Lathe and Bork, 2001). In some bacteria with two copies of *tuf*, deletion of one copy of the gene is not lethal to the bacterium (Hughes, 1990; Zuurmond et al., 1999). It has been postulated that a second copy of this gene (which is mainly conserved in Gram negative bacteria) evolved before the branching of eubacteria (Lathe and Bork, 2001). The cause for the intermittent presence of the second copy of *tuf* within eubacteria has been debated. It has been proposed that the second copy arose by lateral gene transfer, at least within Enterococci (Ke et al., 2000), whilst others argue that lateral gene transfer is unlikely in translation factors and attribute the discontinuous observation of a second *tuf* gene to the theory that it had been randomly lost in some lineages (Lathe and Bork, 2001).

Eukaryotes have two isoforms of EF-Tu known as eEF1A1 and eEF1A2 (Table 1), with each sharing 96% amino acid similarity (Abbas et al., 2015). Both isoforms are also highly expressed representing 1–11% of the total protein expressed (Slobin, 1980; Abbas et al., 2015). Some cells express just one of the eEF1A isoforms, while both are expressed after muscle trauma and in some tumor cell types (Bosutti et al., 2007; Abbas et al., 2015). The number of genes encoding eEF1A varies widely within eukaryotes, from ten in maize to four in rice (Ejiri, 2002).

## Moonlighting Proteins in Bacteria

**FIGURE 2:**
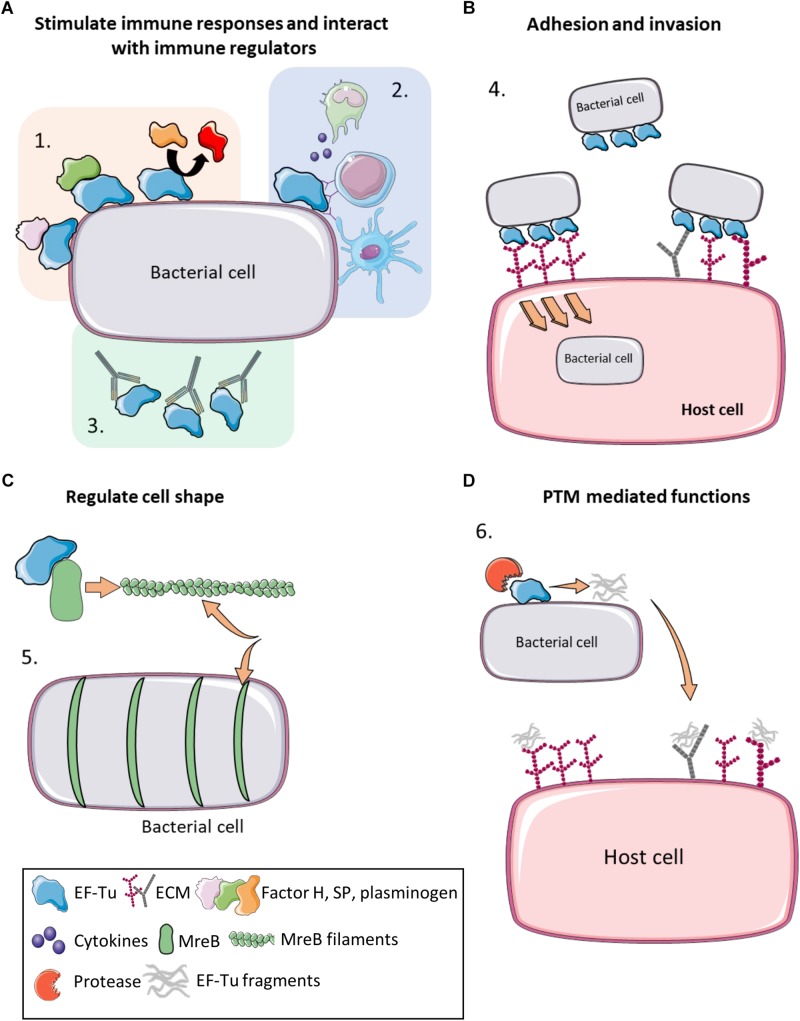
Moonlighting functions of prokaryote EF-Tu. **(A)** (1) EF-Tu binds immune system regulators such as Factor H, substance P and plasminogen (and enhancing its conversion to plasmin), increasing virulence and immune system evasion. (2) EF-Tu stimulates both host innate and humoral immune responses. (3) Antibodies against EF-Tu decrease bacterial load and offer at least partial protection against some bacterial infections. **(B)** (4) EF-Tu binding to fibronectin facilitates invasion into host cells. EF-Tu also binds to other extracellular matrix (ECM) proteins, such as glycosaminoglycans, facilitating adhesion. **(C)** (5) EF-Tu binds MreB and facilitates production of MreB filaments that regulate cell shape. **(D)** (6) EF-Tu undergoes proteolytic processing and EF-Tu fragments also bind ECM proteins. Furthermore, these fragments may act as molecular decoys to help evade immune detection.

There is now overwhelming evidence that proteins with canonical functions in the bacterial cytosol also perform important tasks on the bacterial cell surface (Sanchez et al., 2008; Kainulainen and Korhonen, 2014; Ebner and Götz, 2019). EF-Tu features prominently in many of these studies. Many moonlighting proteins are ancient, highly expressed enzymes, or are proteins that are perform essential roles in glycolysis, respiration and respond to stress (Henderson and Martin, 2011). There is evidence that only a subset of cytosolic proteins can traffic onto the cell because other highly expressed cytosolic proteins are not observed on the cell surface or in extracellular secretions (Vanden Bergh et al., 2013). Mass spectrometry studies have been instrumental in revealing the identities of surface accessible proteins that are not predicted to reside on the cell surface that have canonical functions in the bacterial cytosol (Jeffery, 2005; Robinson et al., 2013; Jarocki et al., 2015; Tacchi et al., 2016; Wang and Jeffery, 2016; Widjaja et al., 2017). The presence of surface-associated moonlighting proteins has been confirmed using florescence and electron microscopy (Bergmann et al., 2001; Candela et al., 2010; Yamaguchi et al., 2010; Robinson et al., 2013; Grundel et al., 2015; Jarocki et al., 2015). It is notable that purified, soluble moonlighting protein fails to associate with the surface when exogenously incubated with bacterial cells (Saad et al., 2009) suggesting that posttranslational modification(s) that occur in the host bacteria and/or passage through the cell membrane may be important events in a proteins ability to moonlight on the cell surface. Another unusual feature of protein moonlighting is that not all strains belonging to the same species present moonlighting proteins on their cell surface. For example, only a subset of pathogenic *E. coli* express surface GAPDH which binds host molecules (Egea et al., 2007). Finally, it is now known that moonlighting proteins are processed on the surface of bacterial pathogens. Processing is expected to increase protein disorder and alter function compared to the full length proteoform (Tacchi et al., 2016). Here we present key studies that describe the salient features that define the diverse moonlighting functions of EF-Tu related to pathogenesis (Figure 2 and Table 2).

**TABLE 2:** List of moonlighting functions published for EF-Tu in prokaryotes.

**Species**	**Moonlighting function**	**Year**	**References**
*Acidovorax avenae*	Rice plants recognize the central amino acids (175-225aa) of EF-Tu as a PAMP	2014	Furukawa et al., 2014
*Acinetobacter baumannii*	Binds fibronectin Binds plasminogen	2012 2015	Dallo et al., 2012 Koenigs et al., 2015
*Actinobacillus seminis*	Binds fibrinogen and fibronectin	2018	Montes-García et al., 2018
*Bacillus anthracis*	Binds plasminogen to evade C3b-dependent innate immunity	2011	Chung et al., 2011
*Bacillus cereus*	Target for Substance P (SP)	2013 2019	Mijouin et al., 2013 N’Diaye et al., 2019
*Bacillus subtilis*	Binds calcium ions Role in cell shape maintenance, colocalizes and modulates MreB filament formation	2009 2010 2015	Dominguez et al., 2009 Defeu Soufo et al., 2010, 2015
*Escherichia coli*	Cleaved in response to phage infection inducing phage exclusion induction *Arabidopsis thaliana* recognizes the first 18 aa of EF-Tu as a PAMP Interacts and modulates MreB filament formation Interacts with DsbA	1994 1998 2000 2004 2005 2014	Bingham et al., 2000 Georgiou et al., 1998 Yu and Snyder, 1994 Kunze et al., 2004 Butland et al., 2005 Premkumar et al., 2014
*Francisella tularensis*	Interacts with THP-1 nucleolin	2008	Barel et al., 2008
*Gallibacterium anatis*	Forms filaments, binds fibronectin and fibrinogen	2017	López-Ochoa et al., 2017
*Helicobacter pylori*	Adheres to THP-1 cells, novel potential adhesion factor	2016	Chiu et al., 2016
*Klebsiella pneumonia*	Virulence factor for Leukopenia caused by *Klebsiella pneumonia*	2014	Liu et al., 2014
*Lactobacillus johnsonii*	Attachment to human intestinal cells and mucins, and participates in host immunomodulation (IL-8 production)	2004	Granato et al., 2004
*Lactobacillus delbrueckii*	Binds mucin	2013	Dhanani and Bagchi, 2013
*Lactobacillus paraplantarum*	Modulates biofilm formation	2017	Liu et al., 2017
*Lactobacillus plantarum*	Adheres to Caco-2 cells Binds mucin Binds actin	2008 2011 2013 2018	Ramiah et al., 2008 Dhanani et al., 2011 Dhanani and Bagchi, 2013 Peng et al., 2018
*Leptospira interrogans serovar Copenhageni*	Binds Factor H and plasminogen (and other ECM)	2013	Wolff et al., 2013
*Listeria monocytogenes*	Binds plasminogen Induces dendritic cell maturation	2004 2016	[Bibr B190][Bibr B141]
*Mycobacterium avium* subsp. *Paratuberculosis*	Binds fibronectin	2014	Viale et al., 2014
*Mycoplasma fermentans*	Interacts with the intracytoplasmic domain of CD21 (EBV/C3d receptor)	2005	Balbo et al., 2005
*Mycoplasma hyopneumoniae*	Fragments bind heparin and fibronectin Binds A594 cells, fetuin, actin, heparin, and plasminogen Binds fibronectin	2016 2017 2018	Tacchi et al., 2016 Widjaja et al., 2017 Yu et al., 2018
*Mycoplasma pneumoniae*	Binds fibronectin Binds A594 cells, fetuin, actin, heparin, and plasminogen	2002 2008 2017	Dallo et al., 2002 Balasubramanian et al., 2008 Widjaja et al., 2017
*Pseudomonas aeruginosa*	Binds Factor H and plasminogen Trimethylation of the lysine allowing binding to platelet-activating receptor Part of the TVISS	2007 2013 2015	Kunert et al., 2007 Barbier et al., 2013 Whitney et al., 2015
*Staphylococcus aureus*	Target for Substance P (SP)	2016	N’Diaye et al., 2016
*Staphylococcus epidermidis*	Target for Substance P (SP)	2016	N’Diaye et al., 2016
*Streptococcus gordonii*	Binds saliva mucin MUC7	2009	Kesimer et al., 2009
*Streptococcus pneumoniae*	Binds Factor H, FHL-1, CFHR1 and plasminogen	2014	Mohan et al., 2014

*Table is arranged based on function, showing different species have evolved the same EF-Tu moonlighting functions. The majority of these moonlighting functions have only been described in the last decade.*

### EF-Tu Is Exposed on the Surface of Bacteria

EF-Tu was first described as having a moonlighting function on the cell surface of *M. pneumoniae* (Dallo et al., 2002). As EF-Tu has now been found on the surface of a wide range of prokaryotes (Table 3), the potential mechanisms behind its extracellular locale shall be summarized here.

**TABLE 3:** Literature reporting the identification of bacterial EF-Tu in non-cytoplasmic locations.

	**References**		
	
**Species**	**Surface exposed^†^**	**Secretome**	**Immunoproteome**
*Actinobacillus seminis*	Montes-García et al., 2018		
*Arsukibacterium ikkense*		Lylloff et al., 2016	
*Bacillus anthracis*		Kim et al., 2014	
*Bacillus cereus*		Clair et al., 2013;	
		Laouami et al., 2014;	
		Voros et al., 2014;	
		Madeira et al., 2015;	
		Omer et al., 2015;	
*Bacteroides fragilis*	Wilson et al., 2015	Wilson et al., 2015	
*Borrelia burgdorferi*			Carrasco et al., 2015
*Brucella abortus*		Jain et al., 2014	
*Burkholderia pseudomallei*		Burtnick et al., 2014	
		Schwarz et al., 2014	
*Caulobacter crescentus*	Cao and Bazemore-Walker, 2014		
*Cellulomonas fimi*		Wakarchuk et al., 2016	
*Cellulomonas flavigena*		Wakarchuk et al., 2016	
*Desulfotomaculum reducens*	Dalla Vecchia et al., 2014		
*Enterococcus faecalis*	Sinnige et al., 2015	Arntzen et al., 2015	
*Escherichia coli*		Boysen et al., 2015	Kudva et al., 2015
*Gallibacterium anatis*	López-Ochoa et al., 2017		
*Haemophilus influnzae*	Thofte et al., 2018		
*Helicobacter pylori*		Chiu et al., 2016	
		Snider et al., 2016	
*Klebsiella pneumonia*			Liu et al., 2014
*Lactobacillus rhamnosus*	Espino et al., 2015		Espino et al., 2015
*Leptospira biflexa*	Wolff et al., 2013;		
	Stewart et al., 2015		
*Leptospira borgpetersenii*	Wolff et al., 2013		
*Leptospira interrogans*	Wolff et al., 2013	Eshghi et al., 2015	
*Leptospira kirschneri*	Wolff et al., 2013		
*Leptospira noguchii*	Wolff et al., 2013		
*Leptospira santarosai*	Wolff et al., 2013		
*Listeria monocytogenes*	Tiong et al., 2015	Rychli et al., 2016	
*Mycobacterium avium* subsp. *paratuberculosis*		Viale et al., 2014	Viale et al., 2014
*Mycobacterium tuberculosis*		Chande et al., 2015	
*Mycoplasma hyopneumoniae*	Tacchi et al., 2016;		
	Yu et al., 2018		
*Mycoplasma myocoides* subsp. *capri*			Churchward et al., 2015
*Mycoplasma pneumoniae*	Widjaja et al., 2017		
*Neisseria meningitidis*		Newcombe et al., 2014	Newcombe et al., 2014
*Propionibacterium freudenreichii*	Le Marechal et al., 2015		
*Pseudomonas aeruginosa*	Kunert et al., 2007	Reales-Calderon et al., 2015	
*Pseudomonas syringae*		Schumacher et al., 2014	
*Roseobacter pomeroyi*		Christie-Oleza et al., 2015a	
*Staphylococcus aureus*		Peton et al., 2014;	Kloppot et al., 2015
		Liew et al., 2015;	
		Mishra and Horswill, 2017	
*Staphylococcus carnosus*		Nega et al., 2015	
*Staphylococcus epidermidis*		Siljamaki et al., 2014	
*Streptococcus gordonii*		Maddi et al., 2014	
*Streptococcus agalactiae*	Yang et al., 2018		
*Streptococcus pneumoniae*	Mohan et al., 2014	Pribyl et al., 2014	
	Pribyl et al., 2014;		
	Jimenez-Munguia et al., 2015		
*Streptococcus thermophilus*	Lecomte et al., 2014		
*Streptomyces scabiei*		Komeil et al., 2014;	
		Padilla-Reynaud et al., 2015	
*Synechococcus* sp.		Christie-Oleza et al., 2015b	
*Tistlia consotensis*		Rubiano-Labrador et al., 2015	
*Vibrio cholerae*		Altindis et al., 2015	
*Vibrio parahaemolyticus*		He et al., 2015	
*Xanthomonas citri* subsp. *citri*		Ferreira et al., 2016	
*Xylella fastidiosa*		Nascimento et al., 2016	

*Articles searched between 2014–2018 identified EF-Tu present on the surface†or in secretions of many bacterial species. Antibodies to this protein were also identified in a number of studies. This table includes data from accessible Supplementary Files. Key search terms used were, but not limited to: exoproteome, secretome, surfacome, surfaceome, immunoproteome, and secretomic. Table excludes any data that was only bioinformatically determined. ^†^Includes surfacome, outer membrane, specific protein analysis under any condition.*

Typically, newly synthesized proteins destined for the cell surface possess either signal peptides or signal motifs that are recognized by transport machinery and translocated through the cytoplasmic membrane (Green and Mecsas, 2016). However, conventionally cytosolic proteins lacking signal sequences, like EF-Tu, have also been identified extracellularly. These proteins, termed non-classically secreted proteins (Wang et al., 2016), can be differentiated from other cytosolic proteins by assessing properties such as amino acid composition and structurally disordered regions (Bendtsen et al., 2005). Indeed, EF-Tu was one protein used to construct the feature-based non-classically secreted protein prediction software SecretomeP (Bendtsen et al., 2005). However, the actual mechanisms behind non-classical protein secretion remain a topic for debate. While there are translocation systems that do not require signal peptides, such as the Holin–Antiholin system, ABC transporters, and a type seven secretion system in Gram-positive bacteria (Götz et al., 2015), these only account for a small portion of non-classically secreted proteins (Wang et al., 2016). Therefore, the presence of cytosolic proteins in extracellular locations is often linked with cell lysis.

There is evidence supporting cytosolic protein excretion through cell lysis, and there is evidence supporting excretion through alternative secretion pathways (reviewed in Wang et al., 2013a). In addition to specific secretion pathways, cytosolic protein excretion can also occur through compromised membrane integrity, translational and osmotic stress, the protein’s biochemical and structural properties, and via membrane vesicles (MVs) (Outer Membrane Vesicles [OMVs] in Gram-negative bacteria) (reviewed in Ebner and Götz, 2019).

Multiple mechanism may contribute to the surface location of EF-Tu; however, there are substantial reports of MVs carrying EF-Tu. Bacterial MVs are nanoparticles produced through processes such as membrane blebbing and endolysin-triggered cell death and contain various membrane and cytosolic proteins, as well as lipopolysaccharides, peptidoglycan and DNA (Turnbull et al., 2016; Toyofuku et al., 2019). MVs are involved in diverse biological processes, including virulence, biofilm development, quorum sensing, horizontal gene transfer, and exportation of cellular components (Toyofuku et al., 2019). EF-Tu is present within MVs derived from Gram-positive bacteria including *Listeria monocytogenes* (Coelho et al., 2019), *Mycobacterium bovis*, *Mycobacterium smegmatis*, *Mycobacterium tuberculosis* (Prados-Rosales et al., 2011), *Staphylococcus aureus* (Lee et al., 2009; Wang et al., 2018), *Streptococcus agalactiae* (Surve et al., 2016), *Streptococcus pnuemoniae* (Olaya-Abril et al., 2014), and *Streptococcus pyogenes* (Resch et al., 2016); Gram-negative bacteria including *Acinetobacter baumannii* (Kwon et al., 2009), *Bacteroides fragilis* (Zakharzhevskaya et al., 2017), *Cronobacter sakazakii* (Alzahrani et al., 2015), *Escherichia coli* (Lee et al., 2007), *Francisella novicida* (Pierson et al., 2011), *Haemophilus influenzae* (Sharpe et al., 2011), *Klebsiella pneumoniae* (Lee et al., 2012), *Neisseria gonorrhoeae* (Pérez-Cruz et al., 2015), *Neisseria meningitidis* (Vipond et al., 2006), and *Pseudomonas aeruginosa* (Choi et al., 2011); and in six *Mycoplasma* species (Gaurivaud et al., 2018). Indeed, EF-Tu was reported as one of the most abundant protein in some of these studies (Lee et al., 2009; Pérez-Cruz et al., 2015; Gaurivaud et al., 2018). Interestingly, several MVs that contain EF-Tu have been reported to increase virulence (Surve et al., 2016), modulate immune responses (Prados-Rosales et al., 2011; Sharpe et al., 2011; Alzahrani et al., 2015), and offer protection to infection via immunization (Vipond et al., 2006; Pierson et al., 2011; Olaya-Abril et al., 2014). As the number of MV-encapsulated proteins varied dramatically within this subset of studies, ranging from 8 in *S. agalactiae* to 416 in *F. novicida*, it is not possible to determine the exact role EF-Tu plays in these processes in most instances. However, in the case of *S. pneumoniae* MVs, which were shown to have high immunogenic capacity and induce protective responses in mice, EF-Tu was one of 15/161 MV proteins that were immunogenic and one of two proteins from which antibodies were generated against in immunized mice (Olaya-Abril et al., 2014).

### EF-Tu Stimulates a Humoral Immune Response and Interacts With Host Immune Regulators

Antibodies against EF-Tu have also been detected in a range of natural infections, including those caused by *Mycoplasma hyopneumoniae* (Pinto et al., 2007), *Chlamydia trachomatis* (Sanchez-Campillo et al., 1999) and *K*. *pneumonia* (Liu et al., 2014). Recombinant EF-Tu (rEF-Tu) from *Mycoplasma ovipneumoniae* induces an immune response in mice, increasing levels of IgG, TNF-α, IFN-γ, IL-12(p70), IL-4, IL-5, and IL-6. Sera from mice immunized with rEF-Tu also reduced *M. ovipneumoniae* growth (Jiang et al., 2016). In *Mycoplasma fermentans*, EF-Tu interacts specifically with the C-terminal 34 amino acids of CD21 in human B lymphoma cells (Balbo et al., 2005). CD21 receptors on the B cells enable the complement system to influence B-cell activation and maturation. The implication is that *Mycoplasma* species are involved in malignancies (reviewed in Vande Voorde et al., 2014) and the interaction between EF-Tu and CD21 may be significant in this regard. EF-Tu from *L. monocytogenes* is the main activator of host dendritic cells (DC) (Mirzaei et al., 2016), antigen presenting cells (APCs) that play a key role in host immune modulation. Activation of DCs is achieved when *L. monocytogenes* interacts with the pattern recognition receptors (PRRs) on the surface of DCs (Mirzaei et al., 2016). These PRRs recognize bacterial proteins known as PAMPS (pathogen-associated molecular pattern). In *L. monocytogenes* EF-Tu was identified as a potent immuno-stimulatory effector in this process indicating that EF-Tu is a candidate for DC maturation-based therapies (Mirzaei et al., 2016).

Recombinant EF-Tu (rEF-Tu) has recently shown promising results as a vaccine candidate against bacterial pathogens. Immunization with rEF-Tu elicited both Th1 and Th2-type responses against *Streptococcus suis* and anti-rEF-Tu sera reduced viable load detection in porcine blood (Feng et al., 2018). Mice immunized with rEF-Tu demonstrated significant protection against lethal challenges with *S. pneumoniae* and increased cytokine, IgG1 and IgG2a, and CD4^+^ T-cell production (Nagai et al., 2019). rEF-Tu mediated protection against *S. pneumoniae* has also been demonstrated in fish (Yang et al., 2018), and partial protection against *H. influenzae* was achieved in mice (Thofte et al., 2018).

Besides stimulating an immune response in mammals (Figure 2A), EF-Tu is also a recognized PAMP in plants (Kunze et al., 2004; Furukawa et al., 2014). EF-Tu is secreted by via unknown mechanisms in soil dwelling, plant–pathogenic bacteria and is recognized by membrane-associated PRRs found on the extracellular surface of root epithelial cells in different plant species. The interaction between PRRs and PAMPS identifies the bacteria as an infectious threat, triggering a signal transduction cascade, that elicits an innate immune response (Zipfel, 2008) that includes the production of reactive-oxygen species and programed cell death (Zipfel, 2008).

Both monocots and dicots use EF-Tu to notify their immune system of an infection. There is however, another level of sophistication to this interaction (Furukawa et al., 2014). Different plant species are known to have evolved recognition mechanisms in their respective PRRs that interact with different regions in the EF-Tu molecule. Rice PRRs recognize the amino acids (aa) 175-225 of EF-Tu, termed EFa50, from the plant pathogenic bacteria *Acidovorax citrulli* (formerly *Acidovorax avenae*) (Furukawa et al., 2014) whereas *Arabidopsis thaliana* recognizes the first 18 aa of EF-Tu from *E. coli* (termed elf18) (Kunze et al., 2004). Although elf18 does not illicit a response to this pathogen in rice plants, engineering the Arabidopsis PRR for elf18 into the rice plant enabled rice to recognize elf18 and respond by increasing resistance to bacterial attack (Lu et al., 2015). This proof-of-concept experiment demonstrated that PRRs can to be engineered into the genomes of different crop species and may be beneficial to the farming and food production industry. Transfer of PRRs has already been demonstrated on food crops such as tomatoes and wheat (Lacombe et al., 2010; Schoonbeek et al., 2015). Earlier structural studies of EF-Tu suggested that the first 12 amino acids of EF-Tu are exposed on the surface and the dodecapeptide can act as a competitive inhibitor of the elf18 elicitor (Kunze et al., 2004). The first 12aa of EF-Tu can also suppress the apoptotic response in plant cells allowing bacterial pathogens sufficient time and nutrient resources to colonize and replicate within plant cells (Igarashi et al., 2013). elf18 and EFa50 do not seem to have similar properties, but more recent structural studies have shown that they do appear to be (at least partially) surface exposed on the EF-Tu molecule (Furukawa et al., 2014). These data indicate that these two PAMPS can interact with PRRs on plant cell surfaces.

EF-Tu has also been shown to bind selectively to neuropeptide hormone substance P (SP) (Mijouin et al., 2013; N’Diaye et al., 2016). SP belongs to the tachykinin family of neuropeptides released by nerve and inflammatory cells (Datar et al., 2004) and is linked to many inflammatory diseases because it binds to the neurokinin 1 receptor (NK-1R) which stimulates pro-inflammatory responses (O’Connor et al., 2004). The inability to trigger NK-1R decreases bacterial clearance and increases death rates in mouse models of infection (Verdrengh and Tarkowski, 2008). SP and/or NK-1R have been linked to disease caused by infectious agents (Douglas et al., 2001; Schwartz et al., 2013), autoimmune disorders (Mantyh et al., 1988), psychological disturbances (Fehder et al., 1997; Herpfer and Lieb, 2005; McLean, 2005; Ebner and Singewald, 2006; Carpenter et al., 2008), cancer (Esteban et al., 2006), atopic dermatitis (Toyoda et al., 2002), and cell proliferation (Goode et al., 2003). EF-Tu from *S. aureus, S. epidermidis*, and *B. cereus* has been shown to bind SP, with an associated increase in virulence and biofilm formation (Mijouin et al., 2013; N’Diaye et al., 2016).

rEF-Tu derived from *Lactobacillus johnsonii* triggers a pro-inflammatory response in HT29 cells and increased IL-8 secretion in the presence of CD14 (Granato et al., 2004). IL-8 is known to increase levels of calcium ions within the cell in which it is expressed (Tuschil et al., 1992; Schorr et al., 1999). In *Bacillus subtilis* EF-Tu is a calcium binding protein (Dominguez et al., 2009). However, the link between calcium and EF-Tu in prokaryotes remains tenuous and further studies are needed to investigate the voracity of this association and its implication in the inflammatory response.

### EF-Tu Has a Role in Adherence to Host Molecules

#### Infection

Adhesion to host cells and molecules is fundamental to pathogenesis in many bacterial species as it facilitates colonization, invasion, and host immune subversion (Stones and Krachler, 2016). In many instances, moonlighting functions for EF-Tu are associated with a role in adherence to a range of host molecules and host cells (Figure 2B). This does not appear to be a trend in specific phylogenetic groups of prokaryotes, as it extends through a range of bacteria. EF-Tu resides on the surface of *Francisella tularensis* where it binds to the RGG domain of nucleolin on the surface of the human monocytic cell line THP-1 (Barel et al., 2008). The HB-19 pseudopeptide irreversibly binds to the RGG domain in the C-terminus of nucleolin (Nisole et al., 1999, 2002) and effectively blocks attachment of *Francisella tularensis* (Barel et al., 2008). Consistent with these experiments, EF-Tu and a 32 kDa cleavage fragment of EF-Tu were recovered during affinity chromatography pull-down experiments using nucleolin as bait (Barel et al., 2008). It is notable that cleavage fragments of EF-Tu have been previously described in the cytoplasm and membrane fraction of *L. monocytogenes* (Archambaud et al., 2005) and more recently on the extracellular surfaces of *S. aureus*, *Mycoplasma hyopneumoniae* and *M. pneumoniae* (Widjaja et al., 2015, 2017). Despite this finding, data describing the cleavage products in *L. monocytogenes* has not been reported but is an interesting observation nonetheless that warrants further investigation.

Fibronectin (Fn) is a key component of the extracellular matrix. It is a glycoprotein that binds to integrins embedded in eukaryote cell membranes and provides support and anchors cells to substrata. Many bacterial pathogens and commensals express adhesins that bind Fn and these interactions can trigger cytoskeletal rearrangements that promote host cell invasion (Massey et al., 2001; Deutscher et al., 2010; Seymour et al., 2010, 2012; Henderson et al., 2011; Bogema et al., 2012; Raymond et al., 2015, 2018). Interestingly, Fn also plays a role as a signaling molecule so, binding Fn may serve other functions for the bacteria, compounding their infectivity (Sandig et al., 2009). Indeed, many bacteria have a repertoire of dedicated, secreted adhesins that target Fn (Henderson et al., 2011).

EF-Tu localizes to both the outer membrane (OM) and outer membrane vesicles (OMV) of *A. baumannii* and binds to DsbA (Premkumar et al., 2014), a protein important in protein folding and maturation (Heras et al., 2009). In its external location in *A. baumannii* EF-Tu directly binds Fn (Dallo et al., 2012). The genome-reduced, human pathogen, *M. pneumoniae* also displays EF-Tu on its cell surface and plays an important role in interactions with Fn (Dallo et al., 2002; Widjaja et al., 2017). Anti-EF-Tu antibodies are able to prevent the binding of *M. pneumoniae* to immobilized Fn demonstrating the specificity of this interaction (Dallo et al., 2002). Binding of EF-Tu is confined to the C-terminal region of EF-Tu, with two Fn-binding regions being identified at amino acid positions 192-292 and 314-394 (Balasubramanian et al., 2008). The specificity of the Fn binding domain between amino acids 314-394 was confirmed when peptides spanning this region blocked the binding capacity of EF-Tu to Fn by 62% (Balasubramanian et al., 2009). Notably, EF-Tu from *Mycoplasma genitalium*, which shares 96% identity with EF-Tu from *M. pneumoniae*, does not bind Fn (Balasubramanian et al., 2009). Experimental comparison between the two sequences identified the residues S343, P345 and T357 to be key in the interaction with Fn (Balasubramanian et al., 2009). However, the Fn-binding *A. baumannii* EF-Tu does not possess these key binding residues identified in *M. pneumoniae* (see Supplementary File), suggesting an alternative Fn-binding mechanism. More recently, we have shown that EF-Tu from *M. pneumoniae* is a multifunctional, adhesive moonlighting protein that can bind fetuin, heparin, actin, as well as to plasminogen, vitronectin, lactoferrin, laminin, and fibrinogen (Widjaja et al., 2017).

The exogenous addition of soluble Fn is known to promote the ability of *Mycobacterium avium* subsp. *paratuberculosis* to attach and invade two epithelial cell lines (Secott et al., 2002) but the identity of bacterial cell surface receptor(s) for Fn were not known. EF-Tu is a surface exposed cell wall protein in *Mycobacterium avium* subspecies *paratuberculosis* (MAP) (Viale et al., 2014). With the importance of Fn to MAP adhesion and invasion (Secott et al., 2002), and as EF-Tu is known to bind Fn in *M. pneumoniae* (Dallo et al., 2002; Balasubramanian et al., 2008) and *A. baumannii* (Dallo et al., 2012), it was investigated for its role as a Fn-binding protein. Knowledge of the Fn-binding regions of EF-Tu from *M. pneumoniae* was used to map putative Fn-binding regions in EF-Tu from *A. baumannii* (Dallo et al., 2012). Although the two previously identified Fn-binding regions (Balasubramanian et al., 2008) only showed 73 and 69% identity respectively in the EF-Tu homolog from MAP, ELISA assays showed that MAP EF-Tu binds Fn in a dose-dependent manner (Viale et al., 2014).

EF-Tu binds to human intestinal cells and mucins in a pH-dependent manner in the probiotic bacterium, *Lactobacillus johnsonii* suggesting a role for EF-Tu in gut colonization (Granato et al., 2004). At pH 5, EF-Tu derived from *L. johnsonii* was able to bind to the human colorectal adenocarcinoma cell line HT29 cells, undifferentiated CACO-2 cells and mucins isolated from HT29-MTX cells (Granato et al., 2004). The ability of EF-Tu to bind mucins extends to other anatomical locations, including the saliva mucin, MUC7 (Kesimer et al., 2009). EF-Tu was identified as one of six proteins to bind this mucin in *Streptococcus gordonii*, a major oral colonizer (Kesimer et al., 2009).

When *H. pylori* is co-cultured with THP-1 cells, expression of EF-Tu is upregulated and secreted and shown to localize to the surface of the human monocytic cell line THP-1 implicating it in host adhesion (Chiu et al., 2016).

**FIGURE 3:**
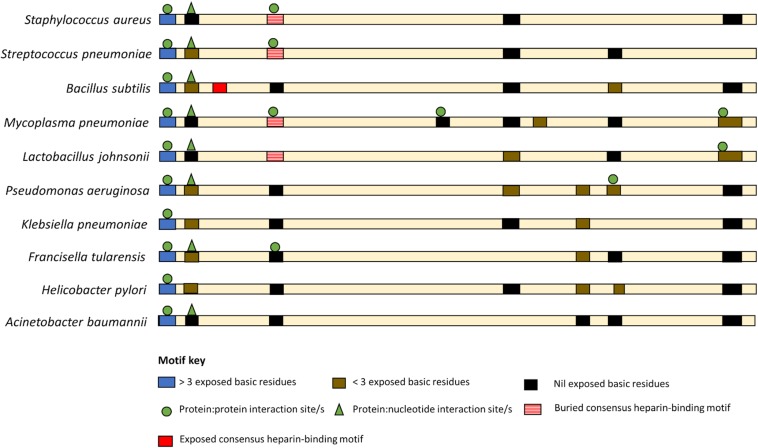
Basic amino acid residue clusters that reside within EF-Tu molecules that moonlight in prokaryotes. An exposed basic amino acid cluster (blue bar) with a putative P:P interaction sites (green circle) is present at the N-termini of moonlighting EF-Tu examples from both Gram positive and Gram-negative bacteria. However, only the Gram-positive bacteria appear to have a consensus heparin binding motif (red bar) and only in *B. subtilis* is this motif predicted to be solvent accessible (solid red bar). The remaining basic residue clusters are predicted to be at least partial buried.

While there are currently no universal moonlighting motifs (Babady et al., 2007), basic residues, both singularly and in clusters, are known to be binding anchors for anionic glycosaminoglycans (Jayaraman et al., 2000), are key in plasminogen (Jarocki et al., 2015), DNA (Ruyechan and Olson, 1992) and actin-binding (Peng et al., 2018), and have been linked with biofilm formation (Shanks et al., 2005; Green et al., 2013). Such basic residue clusters are found throughout the sequences of EF-Tus with reported moonlighting functions (Figure 3 and Supplementary File).

**FIGURE 4:**
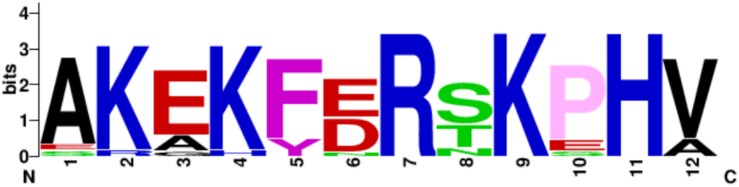
N-termini basic residue cluster logo. The logo is representative of EF-Tu molecules derived from 17 prokaryote species.

A basic residue cluster is located at the N-termini of EF-Tu molecules with known moonlighting functions (Figure 3 and Table 2). This short linear motif (SLiM) has at least three surface exposed basic residues, resides within a region of protein disorder, and possesses predicted protein:protein interaction sites. The arginine and lysine residues in positions 7 and 9 are unconserved in some bacterial species, thus may have arisen from advantageous point mutations (Figure 4).

Interestingly, the moonlighting EF-Tu from *Mycoplasma fermentans* has a unique, highly surface exposed, N-terminal extension that is 39 amino acids in length and possess an additional basic residue cluster at ^31^nKmKgKy^38^ (see Supplementary File). Whether *M. fermentans* EF-Tu harbors moonlighting adhesive capabilities is currently unknown and may warrant future investigation.

Apart from the N-termini, the remaining basic residue clusters observed in EF-Tu molecules that moonlight are at least partially buried, which may impede their ability to bind host molecules. However selective cell surface proteolysis, described in detail in Section “Post-translational Modifications of EF-Tu,” can overcome some of the structural impediments.

#### EF-Tu Binds Innate Immune Effectors

Many bacterial pathogens of medical (Sanderson-Smith et al., 2012) and veterinary (Raymond and Djordjevic, 2015) significance have the ability to bind and activate plasminogen, a process that relies on interactions between basic amino acid residues on surface-accessible bacterial adhesins and kringle domains in plasminogen (Figure 2A). EF-Tu is also utilized by bacteria to dampen the host immune response. The ability of EF-Tu to bind to host complement factors and plasminogen has been demonstrated in *Pseudomonas aeruginosa* (Kunert et al., 2007), *Leptospira* sp. (Wolff et al., 2013), *Streptococcus pneumoniae* (Mohan et al., 2014) *M. pneumoniae* (plasminogen only) (Widjaja et al., 2017) and *Acinetobacter baumannii* (plasminogen only) (Koenigs et al., 2015). The complement system is part of the innate immune system which non-specifically acts to clear the body of infection by lysing bacteria (Lambris et al., 2008). Complement factors bound on the bacterial surface remain active, and plasminogen can be converted to plasmin in its bound state (Kunert et al., 2007; Wolff et al., 2013; Mohan et al., 2014; Raymond and Djordjevic, 2015). This allows the bacteria to regulate and utilize these components for their own gain. By recruiting FH, FH1 and plasminogen the bacteria are able to inactivate C3b via a cleavage event mediated by FH, FH1 and plasmin (Lambris et al., 2008). The C3b complement factor is an important opsonization trigger that binds and labels bacteria ready for opsonization by the host immune system (Lambris et al., 2008). By degrading C3b, the bacteria are able to overcome this aspect of the innate immune system (Lambris et al., 2008). Additionally, the recruitment of plasmin to the bacterial cell surface plays an important role in degrading ECM and facilitating tissue invasion (Lahteenmaki et al., 2000; Bhattacharya et al., 2012). By binding complement factors such as Factor-H, FHL-1 and CFHR1 the bacterium is able to suppress this process and evade the host innate immune response.

Bacterial infections can also lead to a reduction in white blood cell (WBC) counts in infected hosts leading to leukopenia. Isolates of *K. pneumoniae* from patients with leukopenia express higher levels of EF-Tu compared with *K. pneumoniae* isolates from patients with leucocytosis (Liu et al., 2014) suggesting that EF-Tu may be a pathogenicity factor in *K. pneumonia*-based leukopenia (Liu et al., 2014). Interestingly, EF-Tu is upregulated in *Mycobacterium* species that have been phagocytosed by macrophages. The purpose of this upregulation has yet to be determined, but may further support the idea of a role for EF-Tu in bacterial evasion of the host immune system (Monahan et al., 2001).

Not only do bacteria have to defend themselves against the host immune response, but often they must defend themselves against each other. Due to the competitive environment in which bacteria live, they have developed toxins that target other bacterial cells. The Type VI secretion system (T6SS) can deliver toxin molecules into the cytosol of competing bacteria inhabiting the same niche (Pukatzki et al., 2006; Coulthurst, 2013; Cianfanelli et al., 2016). For the T6SS effector molecule in *P. aeruginosa* to enact its toxic effect, it must interact with EF-Tu prior to delivery into the recipient’s cytoplasm (Whitney et al., 2015). The effector molecule and EF-Tu directly bind to each other in members of the *Pseudomonas* genus (Whitney et al., 2015). These observations suggest that EF-Tu has a wide variety of moonlighting functions relating to pathogenesis in different prokaryote species.

#### Cell Shape

Some bacteria can change cell shape as a protective strategy against the immune system. By changing shape, bacteria can become less easily engulfed by phagocytes, and can enhance biofilm formation thereby increasing persistence in the host (van Teeseling et al., 2017). Moreover, major actin-like cytoskeletal proteins, such as MreB, have a role in virulence. For example, in *P. aeruginosa* MreB regulates the type IV pili assemblage (Cowles and Gitai, 2010); in *H. pylori* MreB regulates the secretion of virulence factors (Waidner et al., 2009); and in *Salmonella enterica* serovar Typhimurium a disruption in the *mreB* gene led to downregulation of genes involved in pathogenicity (Bulmer et al., 2012; Doble et al., 2012).

The hypothesis that EF-Tu may interact with the cytoskeleton in prokaryotes is not a new idea, with the concept being introduced as early as the 1970s when EF-Tu was shown to form filaments (Beck, 1979). More recently, the formation of amyloid-like filaments by *Gallibacterium anatis* EF-Tu has been linked to biofilm formation (López-Ochoa et al., 2017). EF-Tu also interacts with MreB in *E. coli* (Butland et al., 2005). MreB forms helical filaments beneath the cell membrane and is essential for regulating cell shape (Jones et al., 2001). As it is well known that eukaryotic EF-Tu (eEF1A) interacts with actin, and influences cell shape, it is conceivable that this moonlighting function also occurs in prokaryotes. In *B. subtilis* and *E. coli* (Defeu Soufo et al., 2010, 2015), EF-Tu modulates the formation of MreB filaments by binding MreB in a ratio of 1:1 (Defeu Soufo et al., 2015) (Figure 2C). One hypothesis suggests a link between EF-Tu cell concentration and cytoskeletal function. By reducing the expression of EF-Tu in the cell, cell shape can be modulated from the typical rod-like appearance to an abnormal cell shape (Defeu Soufo et al., 2010). Alteration of cell shape is due to disruption of the process that places MreB in helical structures beneath the membrane (Defeu Soufo et al., 2010). Further studies investigated whether EF-Tu’s role in the translation mechanism was directly related to the population of EF-Tu molecules that interact with MreB, or whether separate populations of EF-Tu are generated for these alternate roles. Treatment of bacterial cells with kirromycin, which inhibits the release of EF-Tu from the ribosome, failed to interfere with EF-Tu localization in the cytoskeleton or its interactions with MreB filaments (Defeu Soufo et al., 2010). As previous studies have only revealed an interaction between *tufB* with *mreB* (not *tufA*) (Butland et al., 2005), it is tantalizing to consider whether only one of the *tuf* genes is responsible for the alternate function of cytoskeletal integrity. This might suggest that evolution of two *tuf* genes is useful in the delegation of EF-Tu moonlighting roles.

### Post-translational Modifications of EF-Tu

Proteolytic processing is an irreversible post-translational modification (PTM) that can result in a loss, gain or change of function in a protein, as well as in degradation (Turk, 2006). Processing may be a mechanism to unlock moonlighting functions that are inherent in the newly created cleavage fragments via a mechanism similar to ectodomain shedding (Raymond et al., 2013; Tacchi et al., 2016) (Figure 2D). Furthermore, cleavage fragments may serve as competitive inhibitors to host immune cells. Host cytokines, chemokines, enzymes, antimicrobial peptides and growth factors all bind ECM components, including heparin and fibronectin, to control immune responses such as leukocyte emigration through tissue (Gill et al., 2010). By binding to the same ligands as host effector molecules (Kaneider et al., 2007; Krachler and Orth, 2013), bacterial proteins and their cleavage fragments may dampen an immune response. Additionally, cleavage may result in the loss of antigenic epitopes in surface proteins thereby circumventing host immune detection.

Cell surface EF-Tu is proteolytically processed in *M. hyopneumoniae* (Tacchi et al., 2016; Berry et al., 2017; Widjaja et al., 2017). Cleavage fragments of EF-Tu are retained on the cell surface and recovered during affinity chromatography using different host molecules as bait (Tacchi et al., 2016). The cleavage of EF-Tu has recently been demonstrated to be more widespread than previously appreciated with cleavage sites now also mapped in the *M. pneumoniae* and *S. aureus* (Scherl et al., 2005; Plikat et al., 2007; Widjaja et al., 2017). Processing generates fragments that are predicted to be more structural disordered and exposes regions of EF-Tu that are normally inaccessible to the aqueous environment. In particular we have shown that novel SLiMs enriched in positive charges are exposed allowing them to bind to a range of host molecules (Widjaja et al., 2017). Processing has so far been described in bacteria that belong to the low G + C Firmicutes. Many protein:protein and protein:nucleic acid interactions require correctly spaced positive charges derived from lysine, arginine and histidine side chains in short regions of peptide sequence. Amino acids with positively charged side chain residues are encoded by A:T rich triplet codons and members of the low G + C Firmicutes are well suited to employ this as a strategy to expand their functional proteome. Single amino acid substitutions caused by single nucleotide polymorphisms (SNPs) have previously been described as pathogenicity-enhancing (Weissman et al., 2003). Specifically, SNPs in *E. coli* and *S. typhimurium* adhesin genes have led to distinctive pathogenicity-enhanced phenotypes (Sokurenko et al., 1998; Pouttu et al., 1999; Boddicker et al., 2002). SNPs can provide bacteria a selective advantage, leading to niche expansion and ultimately, novel species (Weissman et al., 2003). We propose that the accumulation of positively charged residues via SNPs in SLiMs facilitates binding interactions with diverse host molecules (Widjaja et al., 2017). Processing presents a mechanism to release fragments, each with the potential to expose a different repertoire of SLiMS to the aqueous environment compared with the parent molecule, and generate protein multifunctionality (Widjaja et al., 2017).

Bacterial EF-Tus are also the target for reversible PTMs such as phosphorylation, methylation and acetylation. Phosphorylation of EF-Tu has been identified in *E. coli* (Lippmann et al., 1993), *Thermus thermophiles* (Lippmann et al., 1993), *B. subtilis* (Levine et al., 2006), *Corynebacterium glutamicum* (Bendt et al., 2003), *Streptomyces collinus* (Mikulik and Zhulanova, 1995), *Thiobacillus ferrooxidans* (Seeger et al., 1996), *S. pneumoniae* (Sun et al., 2010), *M. genitalium* (Su et al., 2007), and *M. pneumoniae* (Su et al., 2007). While phosphorylation of EF-Tu lowers its binding affinity to GTP, subsequently reducing protein synthesis, the PTM also inhibits binding by the antibiotic kirromycin, a specific inhibitor of EF-Tu (Archambaud et al., 2005; Sajid et al., 2011). Furthermore, the associated decreased bacterial growth facilitated by EF-Tu phosphorylation has been implicated as an acclimation measure to stress conditions during infection (Archambaud et al., 2005). Similarly, Van Noort et al. (1986) demonstrated that EF-Tu methylation in *E. coli* lowers GTP hydrolysis and suggest a more accurate translation process as a result.

Lysine acetylation and lysine glutarylation has been described in EF-Tu from *Mycobacterium tuberculosis* (Xie et al., 2015, 2016). These modifications can affect protein–protein and protein:nucleic acid interactions and it may be important to the pathogenicity of *M. tuberculosis*, although this is yet to be determined. Evidence for the role of PTMs in EF-Tu moonlighting functions have been described in *P. aeruginosa*, where the trimethylation of the lysine at residue 5, allows EF-Tu to structurally mimic phosphorylcholine (Barbier et al., 2013). This modification means that EF-Tu can specifically bind a platelet-activating receptor, resulting in successful bacterial adhesion (Barbier et al., 2013).

## Concluding Remarks

EF-Tu has evolved to be a multifunctional protein in a wide variety of pathogenic bacteria. While moonlighting functions vary among microbial species there is a common theme for roles in adherence and in immune regulation. The understanding of how this essential and highly expressed protein evolved moonlighting functions is an active area of research and is likely that more diverse and important roles are yet to be discovered.

## Author Contributions

KH and SD conceived and co-wrote the first draft with input from all authors for the final draft. VJ created the figures and contributed significantly to the revised manuscript. All authors contributed to revision, read and approved the submitted version.

## Conflict of Interest

The authors declare that the research was conducted in the absence of any commercial or financial relationships that could be construed as a potential conflict of interest.

## Supplementary Material

FILE S1 Amino acid sequence analysis of EF-Tus with moonlighting functions.

## References

[B1] Abbas W. Kumar A. Herbein G. (2015). The eEF1A proteins: at the crossroads of oncogenesis, apoptosis, and viral infections. *Front. Oncol.* 5:75. 10.3389/fonc.2015.00075 25905039 PMC4387925

[B2] Altindis E. Dong T. Catalano C. Mekalanos J. (2015). Secretome analysis of *Vibrio cholerae* type VI secretion system reveals a new effector-immunity pair. *mBio* 6:e00075-15. 10.1128/mBio.00075-15 25759499 PMC4453574

[B3] Alzahrani H. Winter J. Boocock D. De Girolamo L. Forsythe S. J. (2015). Characterization of outer membrane vesicles from a neonatal meningitic strain of *Cronobacter sakazakii*. *FEMS Microbiol. Lett.* 362:fnv085. 10.1093/femsle/fnv085 26023200

[B4] Archambaud C. Gouin E. Pizarro-Cerda J. Cossart P. Dussurget O. (2005). Translation elongation factor EF-Tu is a target for Stp, a serine-threonine phosphatase involved in virulence of *Listeria monocytogenes*. *Mol. Microbiol.* 56 383–396. 10.1111/j.1365-2958.2005.04551.x 15813732

[B5] Arntzen M. O. Karlskas I. L. Skaugen M. Eijsink V. G. Mathiesen G. (2015). Proteomic Investigation of the response of *Enterococcus faecalis* V583 when cultivated in urine. *PLoS One* 10:e0126694. 10.1371/journal.pone.0126694 25915650 PMC4411035

[B6] Babady N. E. Pang Y.-P. Elpeleg O. Isaya G. (2007). Cryptic proteolytic activity of dihydrolipoamide dehydrogenase. *Proc. Natl. Acad. Sci. U.S.A.* 104 6158–6163. 10.1073/pnas.0610618104 17404228 PMC1851069

[B7] Balasubramanian S. Kannan T. R. Baseman J. B. (2008). The surface-exposed carboxyl region of *Mycoplasma pneumoniae* elongation factor Tu interacts with fibronectin. *Infect. Immun.* 76 3116–3123. 10.1128/IAI.00173-08 18411296 PMC2446705

[B8] Balasubramanian S. Kannan T. R. Hart P. J. Baseman J. B. (2009). Amino acid changes in elongation factor Tu of *Mycoplasma pneumoniae* and *Mycoplasma genitalium* influence fibronectin binding. *Infect. Immun.* 77 3533–3541. 10.1128/IAI.00081-09 19546194 PMC2738037

[B9] Balbo M. Barel M. Lottin-Divoux S. Jean D. Frade R. (2005). Infection of human B lymphoma cells by *Mycoplasma fermentans* induces interaction of its elongation factor with the intracytoplasmic domain of Epstein-Barr virus receptor (gp140, EBV/C3dR, CR2, CD21). *FEMS Microbiol. Lett.* 249 359–366. 10.1016/j.femsle.2005.06.052 16054780

[B10] Baldauf S. L. Palmer J. D. Doolittle W. F. (1996). The root of the universal tree and the origin of eukaryotes based on elongation factor phylogeny. *Proc. Natl. Acad. Sci. U.S.A.* 93 7749–7754. 10.1073/pnas.93.15.7749 8755547 PMC38819

[B11] Barbier M. Owings J. P. Martinez-Ramos I. Damron F. H. Gomila R. Blazquez J. (2013). Lysine trimethylation of EF-Tu mimics platelet-activating factor to initiate *Pseudomonas aeruginosa* pneumonia. *mBio* 4:e00207-13. 10.1128/mBio.00207-13 23653444 PMC3663188

[B12] Barel M. Hovanessian A. G. Meibom K. Briand J. P. Dupuis M. Charbit A. (2008). A novel receptor - ligand pathway for entry of *Francisella tularensis* in monocyte-like THP-1 cells: interaction between surface nucleolin and bacterial elongation factor Tu. *BMC Microbiol.* 8:145. 10.1186/1471-2180-8-145 18789156 PMC2551611

[B13] Beck B. D. (1979). Polymerization of the bacterial elongation factor for protein synthesis, EF-Tu. *Eur. J. Biochem.* 97 495–502. 10.1111/j.1432-1033.1979.tb13137.x 467429

[B14] Bendt A. K. Burkovski A. Schaffer S. Bott M. Farwick M. Hermann T. (2003). Towards a phosphoproteome map of Corynebacterium glutamicum. *Proteomics* 3 1637–1646. 10.1002/pmic.200300494 12923788

[B15] Bendtsen J. D. Kiemer L. Fausbøll A. Brunak S. (2005). Non-classical protein secretion in bacteria. *BMC Microbiol.* 5:58. 10.1186/1471-2180-5-58 16212653 PMC1266369

[B16] Bergmann S. Rohde M. Chhatwal G. S. Hammerschmidt S. (2001). alpha-Enolase of Streptococcus pneumoniae is a plasmin(ogen)-binding protein displayed on the bacterial cell surface. *Mol. Microbiol.* 40 1273–1287. 10.1046/j.1365-2958.2001.02448.x 11442827

[B17] Berry I. J. Jarocki V. M. Tacchi J. L. Raymond B. B. A. Widjaja M. Padula M. P. (2017). N-terminomics identifies widespread endoproteolysis and novel methionine excision in a genome-reduced bacterial pathogen. *Sci. Rep.* 7:11063. 10.1038/s41598-017-11296-9 28894154 PMC5593965

[B18] Bhattacharya S. Ploplis V. A. Castellino F. J. (2012). Bacterial plasminogen receptors utilize host plasminogen system for effective invasion and dissemination. *J. Biomed. Biotechnol.* 2012:482096. 10.1155/2012/482096 23118509 PMC3477821

[B19] Bingham R. Ekunwe S. I. Falk S. Snyder L. Kleanthous C. (2000). The major head protein of bacteriophage T4 binds specifically to elongation factor Tu. *J. Biol. Chem.* 275 23219–23226. 10.1074/jbc.M002546200 10801848

[B20] Boddicker J. D. Ledeboer N. A. Jagnow J. Jones B. D. Clegg S. (2002). Differential binding to and biofilm formation on, HEp-2 cells by *Salmonella enterica* serovar typhimurium is dependent upon allelic variation in the fimH gene of the fim gene cluster. *Mol. Microbiol.* 45 1255–1265. 10.1046/j.1365-2958.2002.03121.x 12207694

[B21] Bogema D. R. Deutscher A. T. Woolley L. K. Seymour L. M. Raymond B. B. A. Tacchi J. L. (2012). Characterization of cleavage events in the multifunctional cilium adhesin Mhp684 (P146) reveals a mechanism by which *Mycoplasma hyopneumoniae* regulates surface topography. *mBio* 3:e00282-11. 10.1128/mBio.00282-11 22493032 PMC3322551

[B22] Bosutti A. Scaggiante B. Grassi G. Guarnieri G. Biolo G. (2007). Overexpression of the elongation factor 1A1 relates to muscle proteolysis and proapoptotic p66(ShcA) gene transcription in hypercatabolic trauma patients. *Metabolism* 56 1629–1634. 10.1016/j.metabol.2007.07.003 17998013

[B23] Boysen A. Borch J. Krogh T. J. Hjerno K. Moller-Jensen J. (2015). SILAC-based comparative analysis of pathogenic *Escherichia coli* secretomes. *J. Microbiol. Methods* 116 66–79. 10.1016/j.mimet.2015.06.015 26143086

[B24] Bulmer D. M. Kharraz L. Grant A. J. Dean P. Morgan F. J. E. Karavolos M. H. (2012). The bacterial cytoskeleton modulates motility, type 3 secretion, and colonization in *Salmonella*. *PLoS Pathog.* 8:e1002500. 10.1371/journal.ppat.1002500 22291596 PMC3266929

[B25] Burtnick M. N. Brett P. J. DeShazer D. (2014). Proteomic analysis of the *Burkholderia pseudomallei* type II secretome reveals hydrolytic enzymes, novel proteins, and the deubiquitinase TssM. *Infect. Immun.* 82 3214–3226. 10.1128/IAI.01739-14 24866793 PMC4136222

[B26] Butland G. Peregrin-Alvarez J. M. Li J. Yang W. Yang X. Canadien V. (2005). Interaction network containing conserved and essential protein complexes in *Escherichia coli*. *Nature* 433 531–537. 10.1038/nature03239 15690043

[B27] Caamano-Antelo S. Fernandez-No I. C. Bohme K. Ezzat-Alnakip M. Quintela-Baluja M. Barros-Velazquez J. (2015). Genetic discrimination of foodborne pathogenic and spoilage *Bacillus* spp. based on three housekeeping genes. *Food Microbiol.* 46 288–298. 10.1016/j.fm.2014.08.013 25475298

[B28] Cacan E. Kratzer J. T. Cole M. F. Gaucher E. A. (2013). Interchanging functionality among homologous elongation factors using signatures of heterotachy. *J. Mol. Evol.* 76 4–12. 10.1007/s00239-013-9540-9 23370546 PMC3585904

[B29] Candela M. Centanni M. Fiori J. Biagi E. Turroni S. Orrico C. (2010). DnaK from *Bifidobacterium animalis* subsp. Lactis is a surface-exposed human plasminogen receptor upregulated in response to bile salts. *Microbiology* 156 1609–1618. 10.1099/mic.0.038307-0 20167618

[B30] Cao Y. Bazemore-Walker C. R. (2014). Proteomic profiling of the surface-exposed cell envelope proteins of *Caulobacter crescentus*. *J. Proteomics* 97 187–194. 10.1016/j.jprot.2013.08.011 23973469

[B31] Carpenter L. L. Bayat L. Moreno F. Kling M. A. Price L. H. Tyrka A. R. (2008). Decreased cerebrospinal fluid concentrations of substance P in treatment-resistant depression and lack of alteration after acute adjunct vagus nerve stimulation therapy. *Psychiatry Res.* 157 123–129. 10.1016/j.psychres.2007.04.016 17976740

[B32] Carrasco S. E. Yang Y. Troxell B. Yang X. Pal U. Yang X. F. (2015). Borrelia burgdorferi elongation factor EF-Tu is an immunogenic protein during Lyme borreliosis. *Emerg. Microbes Infect.* 4:e54. 10.1038/emi.2015.54 26954993 PMC5176084

[B33] Chande A. G. Siddiqui Z. Midha M. K. Sirohi V. Ravichandran S. Rao K. V. (2015). Selective enrichment of mycobacterial proteins from infected host macrophages. *Sci. Rep.* 5:13430. 10.1038/srep13430 26303024 PMC4548221

[B34] Chiu K. H. Wang L. H. Tsai T. T. Lei H. Y. Liao P. C. (2016). Secretomic analysis of host-pathogen interactions reveals that elongation Factor-Tu is a potential adherence factor of *Helicobacter pylori* during pathogenesis. *J. Proteome Res.* 16 264–273. 10.1021/acs.jproteome.6b00584 27764940

[B35] Choi D. S. Kim D. K. Choi S. J. Lee J. Choi J. P. Rho S. (2011). Proteomic analysis of outer membrane vesicles derived from *Pseudomonas aeruginosa*. *Proteomics* 11 3424–3429. 10.1002/pmic.201000212 21751344

[B36] Christie-Oleza J. A. Armengaud J. Guerin P. Scanlan D. J. (2015a). Functional distinctness in the exoproteomes of marine *Synechococcus*. *Env. Microbiol.* 17 3781–3794. 10.1111/1462-2920.12822 25727668 PMC4949707

[B37] Christie-Oleza J. A. Scanlan D. J. Armengaud J. (2015b). “You produce while I clean up”, a strategy revealed by exoproteomics during *Synechococcus*-*Roseobacter* interactions. *Proteomics* 15 3454–3462. 10.1002/pmic.201400562 25728650 PMC4949626

[B38] Chung M.-C. Tonry J. H. Narayanan A. Manes N. P. Mackie R. S. Gutting B. (2011). *Bacillus anthracis* interacts with plasmin(ogen) to evade C3b-dependent innate immunity. *PLoS One* 6:e18119. 10.1371/journal.pone.0018119 21464960 PMC3064659

[B39] Churchward C. P. Rosales R. S. Gielbert A. Dominguez M. Nicholas R. A. Ayling R. D. (2015). Immunoproteomic characterisation of *Mycoplasma mycoides* subspecies capri by mass spectrometry analysis of two-dimensional electrophoresis spots and western blot. *J. Pharm. Pharmacol.* 67 364–371. 10.1111/jphp.12344 25495903

[B40] Cianfanelli F. R. Monlezun L. Coulthurst S. J. (2016). Aim, load, fire: the type VI secretion system, a bacterial nanoweapon. *Trends Microbiol.* 24 51–62. 10.1016/j.tim.2015.10.005 26549582

[B41] Clair G. Lorphelin A. Armengaud J. Duport C. (2013). OhrRA functions as a redox-responsive system controlling toxinogenesis in *Bacillus cereus*. *J. Proteomics* 94 527–539. 10.1016/j.jprot.2013.10.024 24184231

[B42] Coelho C. Brown L. Maryam M. Vij R. Smith D. F. Q. Burnet M. C. (2019). Listeria monocytogenes virulence factors, including listeriolysin O, are secreted in biologically active extracellular vesicles. *J. Biol. Chem.* 294 1202–1217. 10.1074/jbc.RA118.006472 30504226 PMC6349127

[B43] Coulthurst S. J. (2013). The type VI secretion system - a widespread and versatile cell targeting system. *Res. Microbiol.* 164 640–654. 10.1016/j.resmic.2013.03.017 23542428

[B44] Cowles K. N. Gitai Z. (2010). Surface association and the MreB cytoskeleton regulate pilus production, localization and function in *Pseudomonas aeruginosa*. *Mol. Microbiol.* 76 1411–1426. 10.1111/j.1365-2958.2010.07132.x 20398206 PMC3132575

[B45] Dalla Vecchia E. Shao P. P. Suvorova E. Chiappe D. Hamelin R. Bernier-Latmani R. (2014). Characterization of the surfaceome of the metal-reducing bacterium *Desulfotomaculum* reducens. *Front. Microbiol.* 5:432. 10.3389/fmicb.2014.00432 25191310 PMC4137172

[B46] Dallo S. F. Kannan T. R. Blaylock M. W. Baseman J. B. (2002). Elongation factor Tu and E1 beta subunit of pyruvate dehydrogenase complex act as fibronectin binding proteins in *Mycoplasma pneumoniae*. *Mol. Microbiol.* 46 1041–1051. 10.1046/j.1365-2958.2002.03207.x 12421310

[B47] Dallo S. F. Zhang B. Denno J. Hong S. Tsai A. Haskins W. (2012). Association of *Acinetobacter baumannii* EF-Tu with cell surface, outer membrane vesicles, and fibronectin. *ScientificWorldJournal* 2012:128705. 10.1100/2012/128705 22666090 PMC3362023

[B48] Datar P. Srivastava S. Coutinho E. Govil G. (2004). Substance P: structure, function, and therapeutics. *Curr. Top. Med. Chem* 4 75–103. 10.2174/1568026043451636 14754378

[B49] Defeu Soufo H. J. Reimold C. Breddermann H. Mannherz H. G. Graumann P. L. (2015). Translation elongation factor EF-Tu modulates filament formation of actin-like MreB protein in vitro. *J. Mol. Biol.* 427 1715–1727. 10.1016/j.jmb.2015.01.025 25676310

[B50] Defeu Soufo H. J. Reimold C. Linne U. Knust T. Gescher J. Graumann P. L. (2010). Bacterial translation elongation factor EF-Tu interacts and colocalizes with actin-like MreB protein. *Proc. Natl. Acad. Sci. U.S.A.* 107 3163–3168. 10.1073/pnas.0911979107 20133608 PMC2840354

[B51] Deutscher A. T. Jenkins C. Minion F. C. Seymour L. M. Padula M. P. Dixon N. E. (2010). Repeat regions R1 and R2 in the P97 paralogue Mhp271 of Mycoplasma hyopneumoniae bind heparin, fibronectin and porcine cilia. *Mol. Microbiol.* 78 444–458. 10.1111/j.1365-2958.2010.07345.x 20879998

[B52] Dhanani A. S. Bagchi T. (2013). The expression of adhesin EF-Tu in response to mucin and its role in *Lactobacillus adhesion* and competitive inhibition of enteropathogens to mucin. *J. Appl. Microbiol.* 115 546–554. 10.1111/jam.12249 23663754

[B53] Dhanani A. S. Gaudana S. B. Bagchi T. (2011). The ability of *Lactobacillus adhesin* EF-Tu to interfere with pathogen adhesion. *Eur. Food Res. Technol.* 232 777–785. 10.1007/s00217-011-1443-7

[B54] Doble A. C. Bulmer D. M. Kharraz L. Karavolos M. H. Khan C. M. A. (2012). The function of the bacterial cytoskeleton in *Salmonella* pathogenesis. *Virulence* 3 446–449. 10.4161/viru.20993 23076249 PMC3485982

[B55] Dominguez D. C. Lopes R. Palomino J. (2009). *B. subtilis* elongation factor Tu binds Calcium ions. *FASEB J.* 23:LB211.

[B56] Douglas S. D. Ho W. Z. Gettes D. R. Cnaan A. Zhao H. Leserman J. (2001). Elevated substance P levels in HIV-infected men. *AIDS* 15 2043–2045. 10.1097/00002030-200110190-00019 11600835

[B57] Ebner K. Singewald N. (2006). The role of substance P in stress and anxiety responses. *Amino Acids* 31 251–272. 10.1007/s00726-006-0335-9 16820980

[B58] Ebner P. Götz F. (2019). Bacterial excretion of cytoplasmic proteins (ECP): occurrence, mechanism, and function. *Trends Microbiol.* 27 176–187. 10.1016/j.tim.2018.10.006 30442534

[B59] Egea L. Aguilera L. Gimenez R. Sorolla M. A. Aguilar J. Badia J. (2007). Role of secreted glyceraldehyde-3-phosphate dehydrogenase in the infection mechanism of enterohemorrhagic and enteropathogenic *Escherichia coli*: interaction of the extracellular enzyme with human plasminogen and fibrinogen. *Int. J. Biochem. Cell Biol.* 39 1190–1203. 10.1016/j.biocel.2007.03.008 17449317

[B60] Ejiri S. (2002). Moonlighting functions of polypeptide elongation factor 1: from actin bundling to zinc finger protein R1-associated nuclear localization. *Biosci. Biotechnol. Biochem.* 66 1–21. 10.1271/bbb.66.1 11866090

[B61] Eshghi A. Henderson J. Trent M. S. Picardeau M. (2015). *Leptospira interrogans* lpxD homologue is required for thermal acclimatization and virulence. *Infect. Immun.* 83 4314–4321. 10.1128/IAI.00897-15 26283339 PMC4598399

[B62] Espino E. Koskenniemi K. Mato-Rodriguez L. Nyman T. A. Reunanen J. Koponen J. (2015). Uncovering surface-exposed antigens of *Lactobacillus rhamnosus* by cell shaving proteomics and two-dimensional immunoblotting. *J. Proteome Res.* 14 1010–1024. 10.1021/pr501041a 25531588

[B63] Esteban F. Munoz M. Gonzalez-Moles M. A. Rosso M. (2006). A role for substance P in cancer promotion and progression: a mechanism to counteract intracellular death signals following oncogene activation or DNA damage. *Cancer Metastasis Rev.* 25 137–145. 10.1007/s10555-006-8161-9 16680578

[B64] Fehder W. P. Sachs J. Uvaydova M. Douglas S. D. (1997). Substance P as an immune modulator of anxiety. *Neuroimmunomodulation* 4 42–48. 10.1159/000097314 9326744

[B65] Feng L. Niu X. Mei W. Li W. Liu Y. Willias S. P. (2018). Immunogenicity and protective capacity of EF-Tu and FtsZ of *Streptococcus suis* serotype 2 against lethal infection. *Vaccine* 36 2581–2588. 10.1016/j.vaccine.2018.03.079 29627237

[B66] Ferreira R. M. Moreira L. M. Ferro J. A. Soares M. R. Laia M. L. Varani A. M. (2016). Unravelling potential virulence factor candidates in *Xanthomonas citri*. subsp. citri by secretome analysis. *PeerJ* 4:e1734. 10.7717/peerj.1734 26925342 PMC4768671

[B67] Filer D. Furano A. V. (1980). Portions of the gene encoding elongation factor Tu are highly conserved in prokaryotes. *J. Biol. Chem.* 255 728–734. 6985898

[B68] Filer D. Furano A. V. (1981). Duplication of the tuf gene, which encodes peptide chain elongation factor Tu, is widespread in Gram-negative bacteria. *J. Bacteriol.* 148 1006–1011. 6796560 10.1128/jb.148.3.1006-1011.1981 PMC216308

[B69] Furano A. V. (1975). Content of elongation factor Tu in *Escherichia coli*. *Proc. Natl. Acad. Sci. U.S.A.* 72 4780–4784. 10.1073/pnas.72.12.4780 1108000 PMC388815

[B70] Furukawa T. Inagaki H. Takai R. Hirai H. Che F. S. (2014). Two distinct EF-Tu epitopes induce immune responses in rice and *Arabidopsis*. *Mol. Plant Microbe Interact.* 27 113–124. 10.1094/MPMI-10-13-0304-R 24200076

[B71] Gaurivaud P. Ganter S. Villard A. Manso-Silvan L. Chevret D. Boulé C. (2018). *Mycoplasmas* are no exception to extracellular vesicles release: revisiting old concepts. *PLoS One* 13:e0208160. 10.1371/journal.pone.0208160 30485365 PMC6261642

[B72] Georgiou T. Yu Y. N. Ekunwe S. Buttner M. J. Zuurmond A. Kraal B. (1998). Specific peptide-activated proteolytic cleavage of *Escherichia coli* elongation factor Tu. *Proc. Natl. Acad. Sci. U.S.A.* 95 2891–2895. 10.1073/pnas.95.6.2891 9501186 PMC19665

[B73] Gill S. Wight T. N. Frevert C. W. (2010). Proteoglycans: key regulators of pulmonary inflammation and the innate immune response to lung infection. *Anat. Rec. Hoboken N. J.* 2007 968–981. 10.1002/ar.21094 20503391 PMC4121077

[B74] Goode T. O’Connor T. Hopkins A. Moriarty D. O’Sullivan G. C. Collins J. K. (2003). Neurokinin-1 receptor (NK-1R) expression is induced in human colonic epithelial cells by proinflammatory cytokines and mediates proliferation in response to substance P. *J. Cell. Physiol.* 197 30–41. 10.1002/jcp.10234 12942538

[B75] Götz F. Yu W. Dube L. Prax M. Ebner P. (2015). Excretion of cytosolic proteins (ECP) in bacteria. *Int. J. Med. Microbiol.* 305 230–237. 10.1016/j.ijmm.2014.12.021 25596889

[B76] Granato D. Bergonzelli G. E. Pridmore R. D. Marvin L. Rouvet M. Corthesy-Theulaz I. E. (2004). Cell surface-associated elongation factor Tu mediates the attachment of *Lactobacillus johnsonii* NCC533 (La1) to human intestinal cells and mucins. *Infect. Immun.* 72 2160–2169. 10.1128/iai.72.4.2160-2169.2004 15039339 PMC375183

[B77] Green E. Mecsas J. (2016). *Bacterial secretion systems: an overview, in: Virulence Mechanisms of Bacterial Pathogens.* Washington, DC: ASM Press, 215–240.

[B78] Green J. V. Orsborn K. I. Zhang M. Tan Q. K. G. Greis K. D. Porollo A. (2013). Heparin-binding motifs and biofilm formation by *Candida albicans*. *J. Infect. Dis.* 208 1695–1704. 10.1093/infdis/jit391 23904295 PMC4038792

[B79] Grundel A. Friedrich K. Pfeiffer M. Jacobs E. Dumke R. (2015). Subunits of the pyruvate dehydrogenase cluster of *Mycoplasma pneumoniae* are surface-displayed proteins that bind and activate human plasminogen. *PLoS One* 10:e0126600. 10.1371/journal.pone.0126600 25978044 PMC4433191

[B80] He Y. Wang H. Chen L. (2015). Comparative secretomics reveals novel virulence-associated factors of *Vibrio parahaemolyticus*. *Front. Microbiol.* 6:707. 10.3389/fmicb.2015.00707 26236293 PMC4505105

[B81] Henderson B. Martin A. (2011). Bacterial virulence in the moonlight: multitasking bacterial moonlighting proteins are virulence determinants in infectious disease. *Infect. Immun.* 79 3476–3491. 10.1128/IAI.00179-11 21646455 PMC3165470

[B82] Henderson B. Martin A. (2013). Bacterial moonlighting proteins and bacterial virulence. *Curr. Top. Microbiol. Immunol.* 358 155–213. 10.1007/82_2011_188 22143554

[B83] Henderson B. Nair S. Pallas J. Williams M. A. (2011). Fibronectin: a multidomain host adhesin targeted by bacterial fibronectin-binding proteins. *FEMS Microbiol. Rev.* 35 147–200. 10.1111/j.1574-6976.2010.00243.x 20695902

[B84] Heras B. Shouldice S. R. Totsika M. Scanlon M. J. Schembri M. A. Martin J. L. (2009). DSB proteins and bacterial pathogenicity. *Nat. Rev. Microbiol.* 7 215–225. 10.1038/nrmicro2087 19198617

[B85] Herpfer I. Lieb K. (2005). Substance P receptor antagonists in psychiatry: rationale for development and therapeutic potential. *CNS Drugs* 19 275–293. 10.2165/00023210-200519040-00001 15813642

[B86] Huberts D. H. E. W. van der Klei I. J. (2010). Moonlighting proteins: An intriguing mode of multitasking. *Biochim. Biophys. Acta* 1803 520–525. 10.1016/j.bbamcr.2010.01.022 20144902

[B87] Hughes D. (1990). Both genes for EF-Tu in *Salmonella typhimurium* are individually dispensable for growth. *J. Mol. Biol.* 215 41–51. 10.1016/S0022-2836(05)80093-2 2168947

[B88] Igarashi D. Bethke G. Xu Y. Tsuda K. Glazebrook J. Katagiri F. (2013). Pattern-triggered immunity suppresses programmed cell death triggered by fumonisin b1. *PLoS One* 8:e60769. 10.1371/journal.pone.0060769 23560104 PMC3613394

[B89] Iwabe N. Kuma K. Hasegawa M. Osawa S. Miyata T. (1989). Evolutionary relationship of archaebacteria, eubacteria, and eukaryotes inferred from phylogenetic trees of duplicated genes. *Proc. Natl. Acad. Sci. U.S.A.* 86 9355–9359. 10.1073/pnas.86.23.9355 2531898 PMC298494

[B90] Jain S. Kumar S. Dohre S. Afley P. Sengupta N. Alam S. I. (2014). Identification of a protective protein from stationary-phase exoproteome of *Brucella abortus*. *Pathog. Dis.* 70 75–83. 10.1111/2049-632X.12079 23913725

[B91] Jarocki V. M. Santos J. Tacchi J. L. Raymond B. B. A. Deutscher A. T. Jenkins C. (2015). MHJ_0461 is a multifunctional leucine aminopeptidase on the surface of *Mycoplasma hyopneumoniae*. *Open Biol.* 5:140175. 10.1098/rsob.140175 25589579 PMC4313372

[B92] Jayaraman G. Wu C. W. Liu Y. J. Chien K. Y. Fang J. C. Lyu P. C. (2000). Binding of a de novo designed peptide to specific glycosaminoglycans. *FEBS Lett.* 482 154–158. 10.1016/s0014-5793(00)01964-5 11018540

[B93] Jeffery C. J. (1999). Moonlighting proteins. *Trends Biochem. Sci.* 24 8–11. 10.1016/S0968-0004(98)01335-8 10087914

[B94] Jeffery C. J. (2005). Mass spectrometry and the search for moonlighting proteins. *Mass Spectrom. Rev.* 24 772–782. 10.1002/mas.20041 15605385

[B95] Jeffery C. J. (2018). Protein moonlighting: what is it, and why is it important? *Philos. Trans. R. Soc. Lond. B. Biol. Sci.* 373:20160523. 10.1098/rstb.2016.0523 29203708 PMC5717523

[B96] Jiang F. He J. Navarro-Alvarez N. Xu J. Li X. Li P. (2016). Elongation factor Tu and heat shock protein 70 are membrane-associated proteins from *Mycoplasma ovipneumoniae* capable of inducing strong immune response in mice. *PLoS One* 11:e0161170. 10.1371/journal.pone.0161170 27537186 PMC4990256

[B97] Jimenez-Munguia I. van Wamel W. J. Olaya-Abril A. Garcia-Cabrera E. Rodriguez-Ortega M. J. Obando I. (2015). Proteomics-driven design of a multiplex bead-based platform to assess natural IgG antibodies to pneumococcal protein antigens in children. *J. Proteomics* 126 228–233. 10.1016/j.jprot.2015.06.011 26122914

[B98] Jones L. J. Carballido-Lopez R. Errington J. (2001). Control of cell shape in bacteria: helical, actin-like filaments in *Bacillus subtilis*. *Cell* 104 913–922. 11290328 10.1016/s0092-8674(01)00287-2

[B99] Kainulainen V. Korhonen T. K. (2014). Dancing to another tune-adhesive moonlighting proteins in bacteria. *Biol. Basel* 3 178–204. 10.3390/biology3010178 24833341 PMC4009768

[B100] Kaneider N. C. Djanani A. Wiedermann C. J. (2007). Heparan sulfate proteoglycan-involving immunomodulation by cathelicidin antimicrobial peptides LL-37 and PR-39. *ScientificWorldJournal* 7 1832–1838. 10.1100/tsw.2007.285 18040544 PMC5900850

[B101] Ke D. Boissinot M. Huletsky A. Picard F. J. Frenette J. Ouellette M. (2000). Evidence for horizontal gene transfer in evolution of elongation factor Tu in enterococci. *J. Bacteriol.* 182 6913–6920. 10.1128/jb.182.24.6913-6920.2000 11092850 PMC94815

[B102] Kesimer M. Kilic N. Mehrotra R. Thornton D. J. Sheehan J. K. (2009). Identification of salivary mucin MUC7 binding proteins from *Streptococcus gordonii*. *BMC Microbiol.* 9:163. 10.1186/1471-2180-9-163 19671172 PMC2775355

[B103] Kim S. K. Park M. K. Kim S. H. Oh K. G. Jung K. H. Hong C. H. (2014). Identification of stringent response-related and potential serological proteins released from *Bacillus anthracis* overexpressing the RelA/SpoT homolog, Rsh Bant. *Curr. Microbiol.* 69 436–444. 10.1007/s00284-014-0606-8 24838666

[B104] Kjeldgaard M. Nissen P. Thirup S. Nyborg J. (1993). The crystal structure of elongation factor EF-Tu from *Thermus aquaticus* in the GTP conformation. *Structure* 1 35–50. 10.1016/0969-2126(93)90007-4 8069622

[B105] Kloppot P. Selle M. Kohler C. Stentzel S. Fuchs S. Liebscher V. (2015). Microarray-based identification of human antibodies against *Staphylococcus aureus* antigens. *Proteomics Clin. Appl.* 9 1003–1011. 10.1002/prca.201400123 25676254

[B106] Koenigs A. Zipfel P. F. Kraiczy P. (2015). Translation elongation factor Tuf of *Acinetobacter baumannii* is a plasminogen-binding protein. *PLoS One* 10:e0134418. 10.1371/journal.pone.0134418 26230848 PMC4521846

[B107] Komeil D. Padilla-Reynaud R. Lerat S. Simao-Beaunoir A. M. Beaulieu C. (2014). Comparative secretome analysis of *Streptomyces scabiei* during growth in the presence or absence of potato suberin. *Proteome Sci.* 12:35. 10.1186/1477-5956-12-35 25028574 PMC4098958

[B108] Krachler A. M. Orth K. (2013). Targeting the bacteria-host interface: strategies in anti-adhesion therapy. *Virulence* 4 284–294. 10.4161/viru.24606 23799663 PMC3710331

[B109] Kudva I. T. Krastins B. Torres A. G. Griffin R. W. Sheng H. Sarracino D. A. (2015). The *Escherichia coli* O157:H7 cattle immunoproteome includes outer membrane protein A (OmpA), a modulator of adherence to bovine rectoanal junction squamous epithelial (RSE) cells. *Proteomics* 15 1829–1842. 10.1002/pmic.201400432 25643951 PMC4456246

[B110] Kunert A. Losse J. Gruszin C. Huhn M. Kaendler K. Mikkat S. (2007). Immune evasion of the human pathogen *Pseudomonas aeruginosa*: elongation factor Tuf is a factor H and plasminogen binding protein. *J. Immunol.* 179 2979–2988. 10.4049/jimmunol.179.5.2979 17709513

[B111] Kunze G. Zipfel C. Robatzek S. Niehaus K. Boller T. Felix G. (2004). The N terminus of bacterial elongation factor Tu elicits innate immunity in *Arabidopsis* plants. *Plant Cell* 16 3496–3507. 10.1105/tpc.104.026765 15548740 PMC535888

[B112] Kwon S.-O. Gho Y. S. Lee J. C. Kim S. I. (2009). Proteome analysis of outer membrane vesicles from a clinical *Acinetobacter baumannii* isolate. *FEMS Microbiol. Lett.* 297 150–156. 10.1111/j.1574-6968.2009.01669.x 19548894

[B113] Lacombe S. Rougon-Cardoso A. Sherwood E. Peeters N. Dahlbeck D. van Esse H. P. (2010). Interfamily transfer of a plant pattern-recognition receptor confers broad-spectrum bacterial resistance. *Nat. Biotechnol.* 28 365–369. 10.1038/nbt.1613 20231819

[B114] Lahteenmaki K. Kuusela P. Korhonen T. K. (2000). Plasminogen activation in degradation and penetration of extracellular matrices and basement membranes by invasive bacteria. *Methods* 21 125–132. 10.1006/meth.2000.0983 10816373

[B115] Lambris J. D. Ricklin D. Geisbrecht B. V. (2008). Complement evasion by human pathogens. *Nat. Rev. Microbiol.* 6 132–142. 10.1038/nrmicro1824 18197169 PMC2814840

[B116] Laouami S. Clair G. Armengaud J. Duport C. (2014). Proteomic evidences for rex regulation of metabolism in toxin-producing *Bacillus cereus* ATCC 14579. *PLoS One* 9:e107354. 10.1371/journal.pone.0107354 25216269 PMC4162614

[B117] Lathe W. C. Bork P. (2001). Evolution of tuf genes: ancient duplication, differential loss and gene conversion. *FEBS Lett.* 502 113–116. 10.1016/s0014-5793(01)02639-4 11583110

[B118] Le Marechal C. Peton V. Ple C. Vroland C. Jardin J. Briard-Bion V. (2015). Surface proteins of *Propionibacterium freudenreichii* are involved in its anti-inflammatory properties. *J Proteomics* 113 447–461. 10.1016/j.jprot.2014.07.018 25150945

[B119] Lecomte X. Gagnaire V. Briard-Bion V. Jardin J. Lortal S. Dary A. (2014). The naturally competent strain *Streptococcus thermophilus* LMD-9 as a new tool to anchor heterologous proteins on the cell surface. *Microb. Cell Fact.* 13:82. 10.1186/1475-2859-13-82 24902482 PMC4076053

[B120] Lee E.-Y. Bang J. Y. Park G. W. Choi D.-S. Kang J. S. Kim H.-J. (2007). Global proteomic profiling of native outer membrane vesicles derived from *Escherichia coli*. *Proteomics* 7 3143–3153. 10.1002/pmic.200700196 17787032

[B121] Lee E.-Y. Choi D.-Y. Kim D.-K. Kim J.-W. Park J. O. Kim S. (2009). Gram-positive bacteria produce membrane vesicles: Proteomics-based characterization of *Staphylococcus aureus*-derived membrane vesicles. *Proteomics* 9 5425–5436. 10.1002/pmic.200900338 19834908

[B122] Lee J. C. Lee E. J. Lee J. H. Jun S. H. Choi C. W. Kim S. I. (2012). *Klebsiella pneumoniae* secretes outer membrane vesicles that induce the innate immune response. *FEMS Microbiol. Lett.* 331 17–24. 10.1111/j.1574-6968.2012.02549.x 22428779

[B123] Levine A. Vannier F. Absalon C. Kuhn L. Jackson P. Scrivener E. (2006). Analysis of the dynamic *Bacillus subtilis* Ser/Thr/Tyr phosphoproteome implicated in a wide variety of cellular processes. *Proteomics* 6 2157–2173. 10.1002/pmic.200500352 16493705

[B124] Li X. Xing J. Li B. Wang P. Liu J. (2012). Use of tuf as a target for sequence-based identification of Gram-positive cocci of the genus *Enterococcus*, *Streptococcus*, coagulase-negative *Staphylococcus*, and *Lactococcus*. *Ann. Clin. Microbiol. Antimicrob.* 11:31. 10.1186/1476-0711-11-31 23181410 PMC3533577

[B125] Liew Y. K. Awang Hamat R. van Belkum A. Chong P. P. Neela V. (2015). Comparative exoproteomics and host inflammatory response in *Staphylococcus aureus* skin and soft tissue infections, bacteremia, and subclinical colonization. *Clin. Vaccine Immunol.* 22 593–603. 10.1128/CVI.00493-14 25809633 PMC4412950

[B126] Lippmann C. Lindschau C. Vijgenboom E. Schroder W. Bosch L. Erdmann V. A. (1993). Prokaryotic elongation factor Tu is phosphorylated in vivo. *J. Biol. Chem.* 268 601–607. 8416965

[B127] Liu H. Cheng Z. Song W. Wu W. Zhou Z. (2014). Immunoproteomic to analysis the pathogenicity factors in leukopenia caused by *Klebsiella pneumonia* bacteremia. *PLoS One* 9:e110011. 10.1371/journal.pone.0110011 25330314 PMC4199714

[B128] Liu L. Wu R. Zhang J. Shang N. Li P. (2017). D-ribose interferes with quorum sensing to inhibit biofilm formation of *Lactobacillus paraplantarum* L-ZS9. *Front. Microbiol.* 8:1860. 10.3389/fmicb.2017.01860 29018429 PMC5622935

[B129] López-Ochoa J. Montes-García J. F. Vázquez C. Sánchez-Alonso P. Pérez-Márquez V. M. Blackall P. J. (2017). Gallibacterium elongation factor-Tu possesses amyloid-like protein characteristics, participates in cell adhesion, and is present in biofilms. *J. Microbiol.* 55 745–752. 10.1007/s12275-017-7077-0 28865072

[B130] Lu F. Wang H. Wang S. Jiang W. Shan C. Li B. (2015). Enhancement of innate immune system in monocot rice by transferring the dicotyledonous elongation factor Tu receptor EFR. *J. Integr. Plant Biol.* 57 641–652. 10.1111/jipb.12306 25358295

[B131] Lylloff J. E. Hansen L. B. Jepsen M. Sanggaard K. W. Vester J. K. Enghild J. J. (2016). Genomic and exoproteomic analyses of cold- and alkaline-adapted bacteria reveal an abundance of secreted subtilisin-like proteases. *Microb. Biotechnol.* 9 245–256. 10.1111/1751-7915.12343 26834075 PMC4767292

[B132] Maddi A. Haase E. Scannapieco F. (2014). Mass spectrometric analysis of whole secretome and amylase-precipitated secretome proteins from *Streptococcus gordonii*. *J. Proteomics Bioinform.* 7 287–295. 10.4172/jpb.1000331 25605983 PMC4297671

[B133] Madeira J. P. Alpha-Bazin B. Armengaud J. Duport C. (2015). Time dynamics of the *Bacillus cereus* exoproteome are shaped by cellular oxidation. *Front. Microbiol.* 6:342. 10.3389/fmicb.2015.00342 25954265 PMC4406070

[B134] Mantyh C. R. Gates T. S. Zimmerman R. P. Welton M. L. Passaro E. P. Vigna S. R. (1988). Receptor binding sites for substance P, but not substance K or neuromedin K, are expressed in high concentrations by arterioles, venules, and lymph nodules in surgical specimens obtained from patients with ulcerative colitis and Crohn disease. *Proc. Natl. Acad. Sci. U.S.A.* 85 3235–3239. 10.1073/pnas.85.9.3235 2834738 PMC280179

[B135] Massey R. C. Kantzanou M. N. Fowler T. Day N. P. Schofield K. Wann E. R. (2001). Fibronectin-binding protein A of *Staphylococcus aureus* has multiple, substituting, binding regions that mediate adherence to fibronectin and invasion of endothelial cells. *Cell Microbiol.* 3 839–851. 10.1046/j.1462-5822.2001.00157.x 11736995

[B136] McLean S. (2005). Do substance P and the NK1 receptor have a role in depression and anxiety? *Curr. Pharm. Des.* 11 1529–1547. 10.2174/1381612053764779 15892660

[B137] Mignard S. Flandrois J. P. (2007). Identification of *Mycobacterium* using the EF-Tu encoding (tuf) gene and the tmRNA encoding (ssrA) gene. *J. Med. Microbiol.* 56 1033–1041. 10.1099/jmm.0.47105-0 17644709

[B138] Mijouin L. Hillion M. Ramdani Y. Jaouen T. Duclairoir-Poc C. Follet-Gueye M. L. (2013). Effects of a skin neuropeptide (substance p) on cutaneous microflora. *PLoS One* 8:e78773. 10.1371/journal.pone.0078773 24250813 PMC3826737

[B139] Mikulik K. Zhulanova E. (1995). Sequencing of the tuf1 gene and the phosphorylation pattern of EF-Tu1 during development and differentiation in *Streptomyces collinus* producing kirromycin. *Biochem. Biophys. Res. Commun.* 213 454–461. 10.1006/bbrc.1995.2153 7646499

[B140] Min K.-W. Lee S.-H. Baek S. J. (2016). Moonlighting proteins in cancer. *Cancer Lett.* 370 108–116. 10.1016/j.canlet.2015.09.022 26499805 PMC4684795

[B141] Mirzaei R. Saei A. Torkashvand F. Azarian B. Jalili A. Noorbakhsh F. (2016). Identification of proteins derived from *Listeria monocytogenes* inducing human dendritic cell maturation. *Tumour Biol.* 37 10893–10907. 10.1007/s13277-016-4933-1 26886282

[B142] Mishra S. Horswill A. R. (2017). Heparin mimics extracellular DNA in binding to cell surface-localized proteins and promoting *Staphylococcus aureus* biofilm formation. *mSphere* 2:e00135-17. 10.1128/mSphere.00135-17 28656173 PMC5480030

[B143] Mohan S. Hertweck C. Dudda A. Hammerschmidt S. Skerka C. Hallstrom T. (2014). Tuf of *Streptococcus pneumoniae* is a surface displayed human complement regulator binding protein. *Mol. Immunol.* 62 249–264. 10.1016/j.molimm.2014.06.029 25046156

[B144] Monahan I. M. Betts J. Banerjee D. K. Butcher P. D. (2001). Differential expression of mycobacterial proteins following phagocytosis by macrophages. *Microbiology* 147 459–471. 10.1099/00221287-147-2-459 11158363

[B145] Montes-García J. F. Chincoya Martinez D. A. Vaca Pacheco S. Vázquez Cruz C. Sanchez Alonso P. Xicohtencatl Cortes J. (2018). Identification of two adhesins of *Actinobacillus seminis*. *Small Rumin. Res.* 167 100–103. 10.1016/j.smallrumres.2018.08.013

[B146] Nagai K. Domon H. Maekawa T. Hiyoshi T. Tamura H. Yonezawa D. (2019). Immunization with pneumococcal elongation factor Tu enhances serotype-independent protection against *Streptococcus pneumoniae* infection. *Vaccine* 37 160–168. 10.1016/j.vaccine.2018.11.015 30442480

[B147] Nascimento R. Gouran H. Chakraborty S. Gillespie H. W. Almeida-Souza H. O. Tu A. (2016). Corrigendum: the type II secreted Lipase/Esterase LesA is a key virulence factor required for *Xylella fastidiosa* pathogenesis in grapevines. *Sci. Rep.* 6:21575 10.1038/srep21575 PMC4767115 26914974

[B148] N’Diaye A. Mijouin L. Hillion M. Diaz S. Konto-Ghiorghi Y. Percoco G. (2016). Effect of substance P in *Staphylococcus aureus* and *Staphylococcus epidermidis* virulence: implication for skin homeostasis. *Front. Microbiol.* 7:506. 10.3389/fmicb.2016.00506 27148195 PMC4832252

[B149] N’Diaye A. R. Borrel V. Racine P.-J. Clamens T. Depayras S. Maillot O. (2019). Mechanism of action of the moonlighting protein EfTu as a substance P sensor in *Bacillus cereus*. *Sci. Rep.* 9:1304. 10.1038/s41598-018-37506-6 30718605 PMC6361937

[B150] Nega M. Dube L. Kull M. Ziebandt A. K. Ebner P. Albrecht D. (2015). Secretome analysis revealed adaptive and non-adaptive responses of the *Staphylococcus carnosus* femB mutant. *Proteomics* 15 1268–1279. 10.1002/pmic.201400343 25430637 PMC4409834

[B151] Newcombe J. Mendum T. A. Ren C. P. McFadden J. (2014). Identification of the immunoproteome of the meningococcus by cell surface immunoprecipitation and MS. *Microbiology* 160 429–438. 10.1099/mic.0.071829-0 24275101

[B152] Nisole S. Krust B. Callebaut C. Guichard G. Muller S. Briand J. P. (1999). The anti-HIV pseudopeptide HB-19 forms a complex with the cell-surface-expressed nucleolin independent of heparan sulfate proteoglycans. *J. Biol. Chem.* 274 27875–27884. 10.1074/jbc.274.39.27875 10488134

[B153] Nisole S. Said E. A. Mische C. Prevost M. C. Krust B. Bouvet P. (2002). The anti-HIV pentameric pseudopeptide HB-19 binds the C-terminal end of nucleolin and prevents anchorage of virus particles in the plasma membrane of target cells. *J. Biol. Chem.* 277 20877–20886. 10.1074/jbc.M110024200 11919179

[B154] O’Connor T. M. O’Connell J. O’Brien D. I. Goode T. Bredin C. P. Shanahan F. (2004). The role of substance P in inflammatory disease. *J. Cell. Physiol.* 201 167–180. 10.1002/jcp.20061 15334652

[B155] Olaya-Abril A. Prados-Rosales R. McConnell M. J. Martín-Peña R. González-Reyes J. A. Jiménez-Munguía I. (2014). Characterization of protective extracellular membrane-derived vesicles produced by *Streptococcus pneumoniae*. *J. Proteomics* 106 46–60. 10.1016/j.jprot.2014.04.023 24769240

[B156] Omer H. Alpha-Bazin B. Brunet J. L. Armengaud J. Duport C. (2015). Proteomics identifies *Bacillus cereus* EntD as a pivotal protein for the production of numerous virulence factors. *Front. Microbiol.* 6:1004. 10.3389/fmicb.2015.01004 26500610 PMC4595770

[B157] Padilla-Reynaud R. Simao-Beaunoir A. M. Lerat S. Bernards M. A. Beaulieu C. (2015). Suberin regulates the production of cellulolytic enzymes in *Streptomyces scabiei*, the causal agent of potato common scab. *Microbes Env.* 30 245–253. 10.1264/jsme2.ME15034 26330095 PMC4567563

[B158] Peng Z. Vogel R. F. Ehrmann M. A. Xiong T. (2018). Identification and characterization of adhesion proteins in lactobacilli targeting actin as receptor. *Mol. Cell. Probes* 37 60–63. 10.1016/j.mcp.2017.08.002 28823562

[B159] Pérez-Cruz C. Delgado L. López-Iglesias C. Mercade E. (2015). Outer-inner membrane vesicles naturally secreted by gram-negative pathogenic bacteria. *PLoS One* 10:e0116896. 10.1371/journal.pone.0116896 25581302 PMC4291224

[B160] Petit F. M. Serres C. Auer J. (2014). Moonlighting proteins in sperm-egg interactions. *Biochem. Soc. Trans.* 42 1740–1743. 10.1042/BST20140218 25399599

[B161] Peton V. Bouchard D. S. Almeida S. Rault L. Falentin H. Jardin J. (2014). Fine-tuned characterization of *Staphylococcus aureus* Newbould 305, a strain associated with mild and chronic mastitis in bovines. *Vet. Res.* 45:106. 10.1186/s13567-014-0106-7 25316113 PMC4230361

[B162] Pierson T. Matrakas D. Taylor Y. U. Manyam G. Morozov V. N. Zhou W. (2011). Proteomic characterization and functional analysis of outer membrane vesicles of *Francisella novicida* suggests possible role in virulence and use as a vaccine. *J. Proteome Res.* 10 954–967. 10.1021/pr1009756 21138299

[B163] Pinto P. M. Chemale G. de Castro L. A. Costa A. P. M. Kich J. D. Vainstein M. H. (2007). Proteomic survey of the pathogenic *Mycoplasma hyopneumoniae* strain 7448 and identification of novel post-translationally modified and antigenic proteins. *Vet. Microbiol.* 121 83–93. 10.1016/j.vetmic.2006.11.018 17182197

[B164] Plikat U. Voshol H. Dangendorf Y. Wiedmann B. Devay P. Muller D. (2007). From proteomics to systems biology of bacterial pathogens: approaches, tools, and applications. *Proteomics* 7 992–1003. 10.1002/pmic.200600925 17370256

[B165] Polekhina G. Thirup S. Kjeldgaard M. Nissen P. Lippmann C. Nyborg J. (1996). Helix unwinding in the effector region of elongation factor EF-Tu-GDP. *Structure* 4 1141–1151. 10.1016/s0969-2126(96)00122-0 8939739

[B166] Pouttu R. Puustinen T. Virkola R. Hacker J. Klemm P. Korhonen T. K. (1999). Amino acid residue Ala-62 in the FimH fimbrial adhesin is critical for the adhesiveness of meningitis-associated *Escherichia coli* to collagens. *Mol. Microbiol.* 31 1747–1757. 10.1046/j.1365-2958.1999.01311.x 10209747

[B167] Prados-Rosales R. Baena A. Martinez L. R. Luque-Garcia J. Kalscheuer R. Veeraraghavan U. (2011). Mycobacteria release active membrane vesicles that modulate immune responses in a TLR2-dependent manner in mice. *J. Clin. Invest.* 121 1471–1483. 10.1172/JCI44261 21364279 PMC3069770

[B168] Premkumar L. Kurth F. Duprez W. Groftehauge M. K. King G. J. Halili M. A. (2014). Structure of the *Acinetobacter baumannii* dithiol oxidase DsbA bound to elongation factor EF-Tu reveals a novel protein interaction site. *J. Biol. Chem.* 289 19869–19880. 10.1074/jbc.M114.571737 24860094 PMC4106308

[B169] Prezioso S. M. Brown N. E. Goldberg J. B. (2017). Elfamycins: inhibitors of elongation factor-Tu. *Mol. Microbiol.* 106 22–34. 10.1111/mmi.13750 28710887 PMC5701666

[B170] Pribyl T. Moche M. Dreisbach A. Bijlsma J. J. Saleh M. Abdullah M. R. (2014). Influence of impaired lipoprotein biogenesis on surface and exoproteome of *Streptococcus pneumoniae*. *J. Proteome Res.* 13 650–667. 10.1021/pr400768v 24387739

[B171] Pukatzki S. Ma A. T. Sturtevant D. Krastins B. Sarracino D. Nelson W. C. (2006). Identification of a conserved bacterial protein secretion system in *Vibrio cholerae* using the *Dictyostelium* host model system. *Proc. Natl. Acad. Sci. U.S.A.* 103 1528–1533. 10.1073/pnas.0510322103 16432199 PMC1345711

[B172] Ramiah K. van Reenen C. A. Dicks L. M. T. (2008). Surface-bound proteins of Lactobacillus plantarum 423 that contribute to adhesion of Caco-2 cells and their role in competitive exclusion and displacement of *Clostridium sporogenes* and *Enterococcus faecalis*. *Res. Microbiol.* 159 470–475. 10.1016/j.resmic.2008.06.002 18619532

[B173] Raymond B. B. A. Djordjevic S. (2015). Exploitation of plasmin(ogen) by bacterial pathogens of veterinary significance. *Vet. Microbiol.* 178 1–13. 10.1016/j.vetmic.2015.04.008 25937317

[B174] Raymond B. B. A. Jenkins C. Seymour L. M. Tacchi J. L. Widjaja M. Jarocki V. M. (2015). Proteolytic processing of the cilium adhesin MHJ_0194 (P123J) in *Mycoplasma hyopneumoniae* generates a functionally diverse array of cleavage fragments that bind multiple host molecules. *Cell. Microbiol.* 17 425–444. 10.1111/cmi.12377 25293691

[B175] Raymond B. B. A. Tacchi J. L. Jarocki V. M. Minion F. C. Padula M. P. Djordjevic S. P. (2013). P159 from *Mycoplasma hyopneumoniae* binds porcine cilia and heparin and is cleaved in a manner akin to ectodomain shedding. *J. Proteome Res.* 12 5891–5903. 10.1021/pr400903s 24195521

[B176] Raymond B. B. A. Turnbull L. Jenkins C. Madhkoor R. Schleicher I. Uphoff C. C. (2018). *Mycoplasma hyopneumoniae* resides intracellularly within porcine epithelial cells. *Sci. Rep.* 8:17697. 10.1038/s41598-018-36054-3 30523267 PMC6283846

[B177] Reales-Calderon J. A. Corona F. Monteoliva L. Gil C. Martinez J. L. (2015). Quantitative proteomics unravels that the post-transcriptional regulator Crc modulates the generation of vesicles and secreted virulence determinants of *Pseudomonas aeruginosa*. *Data Brief* 4 450–453. 10.1016/j.dib.2015.07.002 26306318 PMC4534582

[B178] Resch U. Tsatsaronis J. A. Le Rhun A. Stübiger G. Rohde M. Kasvandik S. (2016). A two-component regulatory system impacts extracellular membrane-derived vesicle production in group A *Streptococcus*. *mBio* 7:e00207-16. 10.1128/mBio.00207-16 27803183 PMC5090034

[B179] Robinson M. W. Buchtmann K. A. Jenkins C. Tacchi J. L. Raymond B. B. A. To J. (2013). MHJ_0125 is an M42 glutamyl aminopeptidase that moonlights as a multifunctional adhesin on the surface of *Mycoplasma hyopneumoniae*. *Open Biol.* 3:130017. 10.1098/rsob.130017 23594879 PMC3718333

[B180] Rubiano-Labrador C. Bland C. Miotello G. Armengaud J. Baena S. (2015). Salt stress induced changes in the exoproteome of the halotolerant bacterium tistlia consotensis deciphered by proteogenomics. *PLoS One* 10:e0135065. 10.1371/journal.pone.0135065 26287734 PMC4545795

[B181] Ruyechan W. T. Olson J. W. (1992). Surface lysine and tyrosine residues are required for interaction of the major herpes simplex virus type 1 DNA-binding protein with single-stranded DNA. *J. Virol.* 66 6273–6279. 1328667 10.1128/jvi.66.11.6273-6279.1992 PMC240118

[B182] Rychli K. Grunert T. Ciolacu L. Zaiser A. Razzazi-Fazeli E. Schmitz-Esser S. (2016). Exoproteome analysis reveals higher abundance of proteins linked to alkaline stress in persistent *Listeria monocytogenes* strains. *Int. J. Food Microbiol.* 218 17–26. 10.1016/j.ijfoodmicro.2015.11.002 26594790

[B183] Saad N. Urdaci M. Vignoles C. Chaignepain S. Tallon R. Schmitter J. M. (2009). *Lactobacillus plantarum* 299v surface-bound GAPDH: a new insight into enzyme cell walls location. *J. Microbiol. Biotechnol.* 19 1635–1643. 20075631 10.4014/jmb.0902.0102

[B184] Sajid A. Arora G. Gupta M. Singhal A. Chakraborty K. Nandicoori V. K. (2011). Interaction of *Mycobacterium tuberculosis* elongation factor Tu with GTP Is regulated by phosphorylation? *J. Bacteriol.* 193 5347–5358. 10.1128/JB.05469-11 21803988 PMC3187401

[B185] Sanchez B. Bressollier P. Urdaci M. C. (2008). Exported proteins in probiotic bacteria: adhesion to intestinal surfaces, host immunomodulation and molecular cross-talking with the host. *FEMS Immunol. Med. Microbiol.* 54 1–17. 10.1111/j.1574-695X.2008.00454.x 18631181

[B186] Sanchez-Campillo M. Bini L. Comanducci M. Raggiaschi R. Marzocchi B. Pallini V. (1999). Identification of immunoreactive proteins of *Chlamydia trachomatis* by Western blot analysis of a two-dimensional electrophoresis map with patient sera. *Electrophoresis* 20 2269–2279. 10.1002/(SICI)1522-2683(19990801)20:11<2269::AID-ELPS2269>3.0.CO;2-D 10493131

[B187] Sanderson-Smith M. L. De Oliveira D. M. P. Ranson M. McArthur J. D. (2012). Bacterial plasminogen receptors: mediators of a multifaceted relationship. *J. Biomed. Biotechnol.* 2012:272148. 10.1155/2012/272148 23118502 PMC3478875

[B188] Sandig H. McDonald J. Gilmour J. Arno M. Lee T. H. Cousins D. J. (2009). Fibronectin is a TH1-specific molecule in human subjects. *J. Allergy Clin. Immunol.* 124 528.e5–535.e5. 10.1016/j.jaci.2009.04.036 19541353

[B189] Sasikumar A. N. Perez W. B. Kinzy T. G. (2012). The many roles of the eukaryotic elongation factor 1 complex. *Wiley Interdiscip. Rev. RNA* 3 543–555. 10.1002/wrna.1118 22555874 PMC3374885

[B190] Schaumburg J. Diekmann O. Hagendorff P. Bergmann S. Rohde M. Hammerschmidt S. (2004). The cell wall subproteome of *Listeria monocytogenes*. *Proteomics* 4 2991–3006. 10.1002/pmic.200400928 15378750

[B191] Scherl A. Francois P. Bento M. Deshusses J. M. Charbonnier Y. Converset V. (2005). Correlation of proteomic and transcriptomic profiles of *Staphylococcus aureus* during the post-exponential phase of growth. *J. Microbiol. Methods* 60 247–257. 10.1016/j.mimet.2004.09.017 15590099

[B192] Schoonbeek H.-J. Wang H.-H. Stefanato F. L. Craze M. Bowden S. Wallington E. (2015). Arabidopsis EF-Tu receptor enhances bacterial disease resistance in transgenic wheat. *New Phytol.* 206 606–613. 10.1111/nph.13356 25760815

[B193] Schorr W. Swandulla D. Zeilhofer H. U. (1999). Mechanisms of IL-8-induced Ca2+ signaling in human neutrophil granulocytes. *Eur. J. Immunol.* 29 897–904. 10092093 10.1002/(SICI)1521-4141(199903)29:03<897::AID-IMMU897>3.0.CO;2-5

[B194] Schumacher J. Waite C. J. Bennett M. H. Perez M. F. Shethi K. Buck M. (2014). Differential secretome analysis of *Pseudomonas* syringae pv tomato using gel-free MS proteomics. *Front. Plant Sci.* 5:242. 10.3389/fpls.2014.00242 25071788 PMC4082315

[B195] Schwartz L. Spitsin S. V. Meshki J. Tuluc F. Douglas S. D. Wolfe J. H. (2013). Substance P enhances HIV-1 infection in human fetal brain cell cultures expressing full-length neurokinin-1 receptor. *J. Neurovirol.* 19 219–227. 10.1007/s13365-013-0166-x 23765222 PMC3719168

[B196] Schwarz S. Singh P. Robertson J. D. LeRoux M. Skerrett S. J. Goodlett D. R. (2014). VgrG-5 is a *Burkholderia* type VI secretion system-exported protein required for multinucleated giant cell formation and virulence. *Infect. Immun.* 82 1445–1452. 10.1128/IAI.01368-13 24452686 PMC3993412

[B197] Secott T. E. Lin T. L. Wu C. C. (2002). Fibronectin attachment protein is necessary for efficient attachment and invasion of epithelial cells by *Mycobacterium avium* subsp. paratuberculosis. *Infect Immun* 70 2670–2675. 11953410 10.1128/IAI.70.5.2670-2675.2002 PMC127902

[B198] Seeger M. Osorio G. Jerez C. A. (1996). Phosphorylation of GroEL, DnaK and other proteins from *Thiobacillus ferrooxidans* grown under different conditions. *FEMS Microbiol. Lett.* 138 129–134. 9026439 10.1111/j.1574-6968.1996.tb08145.x

[B199] Seymour L. M. Deutscher A. T. Jenkins C. Kuit T. A. Falconer L. Minion F. C. (2010). A processed multidomain *Mycoplasma hyopneumoniae* adhesin binds fibronectin, plasminogen, and swine respiratory cilia. *J. Biol. Chem.* 285 33971–33978. 10.1074/jbc.M110.104463 20813843 PMC2962497

[B200] Seymour L. M. Jenkins C. Deutscher A. T. Raymond B. B. A. Padula M. P. Tacchi J. L. (2012). Mhp182 (P102) binds fibronectin and contributes to the recruitment of plasmin(ogen) to the *Mycoplasma hyopneumoniae* cell surface. *Cell. Microbiol.* 14 81–94. 10.1111/j.1462-5822.2011.01702.x 21951786

[B201] Shanks R. M. Q. Donegan N. P. Graber M. L. Buckingham S. E. Zegans M. E. Cheung A. L. (2005). Heparin stimulates *Staphylococcus aureus* biofilm formation. *Infect. Immun.* 73 4596–4606. 10.1128/IAI.73.8.4596-4606.2005 16040971 PMC1201187

[B202] Sharpe S. W. Kuehn M. J. Mason K. M. (2011). Elicitation of epithelial cell-derived immune effectors by outer membrane vesicles of nontypeable *Haemophilus influenzae*? *Infect. Immun.* 79 4361–4369. 10.1128/IAI.05332-11 21875967 PMC3257905

[B203] Shin J. H. Cho E. J. Lee J. Y. Yu J. Y. Kang Y. H. (2009). Novel diagnostic algorithm using tuf gene amplification and restriction fragment length polymorphism is promising tool for identification of nontuberculous mycobacteria. *J. Microbiol. Biotechnol.* 19 323–330. 19349759 10.4014/jmb.0804.267

[B204] Siljamaki P. Varmanen P. Kankainen M. Pyorala S. Karonen T. Iivanainen A. (2014). Comparative proteome profiling of bovine and human *Staphylococcus epidermidis* strains for screening specifically expressed virulence and adaptation proteins. *Proteomics* 14 1890–1894. 10.1002/pmic.201300275 24909406

[B205] Sinnige J. C. de Been M. Zhou M. Bonten M. J. Willems R. J. Top J. (2015). Growth condition-dependent cell surface proteome analysis of *Enterococcus faecium*. *Proteomics* 15 3806–3814. 10.1002/pmic.201500138 26316380

[B206] Slobin L. I. (1980). The role of eucaryotic factor Tu in protein synthesis. The measurement of the elongation factor Tu content of rabbit reticulocytes and other mammalian cells by a sensitive radioimmunoassay. *Eur. J. Biochem.* 110 555–563. 7192214 10.1111/j.1432-1033.1980.tb04898.x

[B207] Snider C. A. Voss B. J. McDonald W. H. Cover T. L. (2016). Growth phase-dependent composition of the *Helicobacter pylori* exoproteome. *J. Proteomics* 130 94–107. 10.1016/j.jprot.2015.08.025 26363098 PMC4640983

[B208] Sokurenko E. V. Chesnokova V. Dykhuizen D. E. Ofek I. Wu X.-R. Krogfelt K. A. (1998). Pathogenic adaptation of *Escherichia coli* by natural variation of the FimH adhesin. *Proc. Natl. Acad. Sci. U.S.A.* 95 8922–8926. 10.1073/pnas.95.15.8922 9671780 PMC21178

[B209] Sprinzl M. (1994). Elongation factor Tu: a regulatory GTPase with an integrated effector. *Trends Biochem. Sci.* 19 245–250. 8073502 10.1016/0968-0004(94)90149-x

[B210] Stewart P. E. Carroll J. A. Olano L. R. Sturdevant D. E. Rosa P. A. (2015). Multiple posttranslational modifications of *Leptospira biflexa* proteins as revealed by proteomic analysis. *Appl. Environ. Microbiol.* 82 1183–1195. 10.1128/AEM.03056-15 26655756 PMC4751834

[B211] Stones D. H. Krachler A. M. (2016). Against the tide: the role of bacterial adhesion in host colonization. *Biochem. Soc. Trans.* 44 1571–1580. 10.1042/BST20160186 27913666 PMC5134996

[B212] Su H. C. Hutchison C. A. Giddings M. C. (2007). Mapping phosphoproteins in *Mycoplasma genitalium* and *Mycoplasma pneumoniae*. *BMC Microbiol.* 7:63. 10.1186/1471-2180-7-63 17605819 PMC1947986

[B213] Sun X. Ge F. Xiao C. L. Yin X. F. Ge R. Zhang L. H. (2010). Phosphoproteomic analysis reveals the multiple roles of phosphorylation in pathogenic bacterium *Streptococcus pneumoniae*. *J. Proteome Res.* 9 275–282. 10.1021/pr900612v 19894762

[B214] Surve M. V. Anil A. Kamath K. G. Bhutda S. Sthanam L. K. Pradhan A. (2016). Membrane vesicles of Group B *Streptococcus* disrupt feto-maternal barrier leading to preterm birth. *PLoS Pathog.* 12:e1005816. 10.1371/journal.ppat.1005816 27583406 PMC5008812

[B215] Tacchi J. L. Raymond B. B. A. Haynes P. A. Berry I. J. Widjaja M. Bogema D. R. (2016). Post-translational processing targets functionally diverse proteins in *Mycoplasma hyopneumoniae*. *Open Biol.* 6:150210. 10.1098/rsob.150210 26865024 PMC4772806

[B216] Thofte O. Su Y.-C. Brant M. Littorin N. Duell B. L. Alvarado V. (2018). EF-Tu From Non-typeable *Haemophilus influenzae* is an immunogenic surface-exposed protein targeted by bactericidal antibodies. *Front. Immunol.* 9:2910. 10.3389/fimmu.2018.02910 30619274 PMC6305414

[B217] Tiong H. K. Hartson S. Muriana P. M. (2015). Comparison of five methods for direct extraction of surface proteins from *Listeria monocytogenes* for proteomic analysis by orbitrap mass spectrometry. *J. Microbiol. Methods* 110 54–60. 10.1016/j.mimet.2015.01.004 25578509

[B218] Toyoda M. Nakamura M. Makino T. Hino T. Kagoura M. Morohashi M. (2002). Nerve growth factor and substance P are useful plasma markers of disease activity in atopic dermatitis. *Br. J. Dermatol.* 147 71–79. 12100187 10.1046/j.1365-2133.2002.04803.x

[B219] Toyofuku M. Nomura N. Eberl L. (2019). Types and origins of bacterial membrane vesicles. *Nat. Rev. Microbiol.* 17 13–24. 10.1038/s41579-018-0112-2 30397270

[B220] Turk B. (2006). Targeting proteases: successes, failures and future prospects. *Nat. Rev. Drug Discov.* 5 785–799. 10.1038/nrd2092 16955069

[B221] Turnbull L. Toyofuku M. Hynen A. L. Kurosawa M. Pessi G. Petty N. K. (2016). Explosive cell lysis as a mechanism for the biogenesis of bacterial membrane vesicles and biofilms. *Nat. Commun.* 7:11220. 10.1038/ncomms11220 27075392 PMC4834629

[B222] Tuschil A. Lam C. Haslberger A. Lindley I. (1992). Interleukin-8 stimulates calcium transients and promotes epidermal cell proliferation. *J. Invest. Dermatol.* 99 294–298. 1512465 10.1111/1523-1747.ep12616634

[B223] Van Noort J. M. V. Kraal B. Sinjorgo K. M. C. Persoon N. L. M. Johanns E. S. D. Bosch L. (1986). Methylation in vivo of elongation factor EF-Tu at lysine-56 decreases the rate of tRNA-dependent GTP hydrolysis. *Eur. J. Biochem.* 160 557–561. 10.1111/j.1432-1033.1986.tb10074.x 3096728

[B224] van Teeseling M. C. F. de Pedro M. A. Cava F. (2017). Determinants of bacterial morphology: from fundamentals to possibilities for antimicrobial targeting. *Front. Microbiol.* 8:1264. 10.3389/fmicb.2017.01264 28740487 PMC5502672

[B225] Vande Voorde J. Balzarini J. Liekens S. (2014). Mycoplasmas and cancer: focus on nucleoside metabolism. *EXCLI J* 13 300–322. 26417262 PMC4464442

[B226] Vanden Bergh P. Heller M. Braga-Lagache S. Frey J. (2013). The *Aeromonas salmonicida* subsp. salmonicida exoproteome: global analysis, moonlighting proteins and putative antigens for vaccination against furunculosis. *Proteome Sci.* 11 1–12. 10.1186/1477-5956-11-44 24127837 PMC3826670

[B227] Verdrengh M. Tarkowski A. (2008). The impact of substance P signalling on the development of experimental staphylococcal sepsis and arthritis. *Scand. J. Immunol.* 67 253–259. 10.1111/j.1365-3083.2007.02065.x 18226012

[B228] Viale M. N. Echeverria-Valencia G. Romasanta P. Mon M. L. Fernandez M. Malchiodi E. (2014). Description of a novel adhesin of *Mycobacterium avium* subsp. paratuberculosis. *Biomed. Res. Int.* 2014:729618. 10.1155/2014/729618 25136616 PMC4130151

[B229] Vijgenboom E. Woudt L. P. Heinstra P. W. Rietveld K. van Haarlem J. van Wezel G. P. (1994). Three tuf-like genes in the kirromycin producer *Streptomyces ramocissimus*. *Microbiology* 140(Pt 4), 983–998. 8012612 10.1099/00221287-140-4-983

[B230] Vipond C. Suker J. Jones C. Tang C. Feavers I. M. Wheeler J. X. (2006). Proteomic analysis of a meningococcal outer membrane vesicle vaccine prepared from the group B strain NZ98/254. *Proteomics* 6 3400–3413. 10.1002/pmic.200500821 16645985

[B231] Voros A. Simm R. Slamti L. McKay M. J. Hegna I. K. Nielsen-LeRoux C. (2014). SecDF as part of the Sec-translocase facilitates efficient secretion of *Bacillus cereus* toxins and cell wall-associated proteins. *PLoS One* 9:e103326. 10.1371/journal.pone.0103326 25083861 PMC4118872

[B232] Waidner B. Specht M. Dempwolff F. Haeberer K. Schaetzle S. Speth V. (2009). A Novel system of cytoskeletal elements in the human pathogen *Helicobacter* pylori. *PLoS Pathog.* 5:e1000669. 10.1371/journal.ppat.1000669 19936218 PMC2776988

[B233] Wakarchuk W. W. Brochu D. Foote S. Robotham A. Saxena H. Erak T. (2016). Proteomic analysis of the secretome of *Cellulomonas* fimi ATCC 484 and *Cellulomonas* flavigena ATCC 482. *PLoS One* 11:e0151186. 10.1371/journal.pone.0151186 26950732 PMC4780727

[B234] Wang G. Chen H. Xia Y. Cui J. Gu Z. Song Y. (2013a). How are the non-classically secreted bacterial proteins released into the extracellular milieu? *Curr. Microbiol.* 67 688–695. 10.1007/s00284-013-0422-6 23963513

[B235] Wang G. Xia Y. Cui J. Gu Z. Song Y. Chen Y. Q. (2013b). The roles of moonlighting proteins in bacteria. *Curr. Issues Mol. Biol.* 16 15–22. 23872606

[B236] Wang G. Xia Y. Song X. Ai L. (2016). Common non-classically secreted bacterial proteins with experimental evidence. *Curr. Microbiol.* 72 102–111. 10.1007/s00284-015-0915-6 26429784

[B237] Wang W. Jeffery C. J. (2016). An analysis of surface proteomics results reveals novel candidates for intracellular/surface moonlighting proteins in bacteria. *Mol. Biosyst.* 2 1420–1431. 10.1039/c5mb00550g 26938107

[B238] Wang X. Thompson C. D. Weidenmaier C. Lee J. C. (2018). Release of *Staphylococcus aureus* extracellular vesicles and their application as a vaccine platform. *Nat. Commun.* 9:1379. 10.1038/s41467-018-03847-z 29643357 PMC5895597

[B239] Weissman S. J. Moseley S. L. Dykhuizen D. E. Sokurenko E. V. (2003). Enterobacterial adhesins and the case for studying SNPs in bacteria. *Trends Microbiol.* 11 115–117. 12648942 10.1016/s0966-842x(03)00010-6

[B240] Whitney J. C. Quentin D. Sawai S. LeRoux M. Harding B. N. Ledvina H. E. (2015). An interbacterial NAD(P)(+) glycohydrolase toxin requires elongation factor Tu for delivery to target cells. *Cell* 163 607–619. 10.1016/j.cell.2015.09.027 26456113 PMC4624332

[B241] Widjaja M. Berry I. J. Pont E. J. Padula M. P. Djordjevic S. P. (2015). P40 and P90 from Mpn142 are targets of multiple processing events on the surface of *Mycoplasma pneumoniae*. *Proteomes* 3 512–537. 10.3390/proteomes3040512 28248283 PMC5217387

[B242] Widjaja M. Harvey K. L. Hagemann L. Berry I. J. Jarocki V. M. Raymond B. B. A. (2017). Elongation factor Tu is a multifunctional and processed moonlighting protein. *Sci. Rep.* 7:11227. 10.1038/s41598-017-10644-z 28894125 PMC5593925

[B243] Wilson M. M. Anderson D. E. Bernstein H. D. (2015). Analysis of the outer membrane proteome and secretome of *Bacteroides fragilis* reveals a multiplicity of secretion mechanisms. *PLoS One* 10:e0117732. 10.1371/journal.pone.0117732 25658944 PMC4319957

[B244] Wolf H. Chinali G. Parmeggiani A. (1974). Kirromycin, an inhibitor of protein biosynthesis that acts on elongation factor Tu. *Proc. Natl. Acad. Sci. U.S.A.* 71 4910–4914. 4373734 10.1073/pnas.71.12.4910 PMC434009

[B245] Wolff D. G. Castiblanco-Valencia M. M. Abe C. M. Monaris D. Morais Z. M. Souza G. O. (2013). Interaction of Leptospira elongation factor Tu with plasminogen and complement factor H: a metabolic leptospiral protein with moonlighting activities. *PLoS One* 8:e81818. 10.1371/journal.pone.0081818 24312361 PMC3842364

[B246] Xie L. Wang G. Yu Z. Zhou M. Li Q. Huang H. (2016). Proteome-wide lysine glutarylation profiling of the *Mycobacterium tuberculosis* H37Rv. *J. Proteome Res.* 15 1379–1385. 10.1021/acs.jproteome.5b00917 26903315

[B247] Xie L. Wang X. Zeng J. Zhou M. Duan X. Li Q. (2015). Proteome-wide lysine acetylation profiling of the human pathogen *Mycobacterium tuberculosis*. *Int. J. Biochem. Cell Biol.* 59 193–202. 10.1016/j.biocel.2014.11.010 25456444

[B248] Yamaguchi M. Ikeda R. Nishimura M. Kawamoto S. (2010). Localization by scanning immunoelectron microscopy of triosephosphate isomerase, the molecules responsible for contact-mediated killing of *Cryptococcus*, on the surface of *Staphylococcus*. *Microbiol. Immunol.* 54 368–370. 10.1111/j.1348-0421.2010.00225.x 20536736

[B249] Yang Q. Liu J. Wang K. Liu T. Zhu L. He S. (2018). Evaluation of immunogenicity and protective efficacy of the elongation factor Tu against *Streptococcus agalactiae* in tilapia. *Aquaculture* 492 184–189. 10.1016/j.aquaculture.2018.03.056

[B250] Yoon J.-H. Ryu J. Baek S. J. (2018). Moonlighting activity of secreted inflammation-regulatory proteins. *Yonsei Med. J.* 59 463–469. 10.3349/ymj.2018.59.4.463 29749128 PMC5949287

[B251] Yu Y. Wang H. Wang J. Feng Z. Wu M. Liu B. (2018). Elongation factor thermo unstable (EF-Tu) moonlights as an adhesin on the surface of *Mycoplasma hyopneumoniae* by binding to fibronectin. *Front. Microbiol.* 9:974. 10.3389/fmicb.2018.00974 29867877 PMC5962738

[B252] Yu Y. T. Snyder L. (1994). Translation elongation factor Tu cleaved by a phage-exclusion system. *Proc. Natl. Acad. Sci. U.S.A.* 91 802–806. 8290603 10.1073/pnas.91.2.802 PMC43037

[B253] Zakharzhevskaya N. B. Vanyushkina A. A. Altukhov I. A. Shavarda A. L. Butenko I. O. Rakitina D. V. (2017). Outer membrane vesicles secreted by pathogenic and nonpathogenic *Bacteroides fragilis* represent different metabolic activities. *Sci. Rep.* 7:5008. 10.1038/s41598-017-05264-6 28694488 PMC5503946

[B254] Zipfel C. (2008). Pattern-recognition receptors in plant innate immunity. *Curr. Opin. Immunol.* 20 10–16. 10.1016/j.coi.2007.11.003 18206360

[B255] Zuurmond A.-M. Rundlöf A.-K. Kraal B. (1999). Either of the chromosomal *tuf* genes of *E. coli* K-12 can be deleted without loss of cell viability. *Mol. Gen. Genet.* 260 603–607. 10.1007/s004380050934 9928940

