# Supplementary material for: The Diverse Functional Roles of Elongation Factor Tu (EF-Tu) in Microbial Pathogenesis
Source: Front Microbiol. 2019 Oct 24;10:2351. doi: 10.3389/fmicb.2019.02351 (PMC6822514; doi:10.3389/fmicb.2019.02351)
Supplement: FILE S1 — Amino acid sequence analysis of EF-Tus with moonlighting functions. [file Data_Sheet_1.PDF]

●Protein:Protein interaction site △ Nucleotide interaction site ■ α helix ➡ β strand ⇩ Exposed reside

Basic residue cluster **Unconserved residue** *Disordered region*

```
SP|P23568|EFTU_MYCPN      ---------------------------------------MAREKFDRSKPHVNVGTIGHI     21
TR|C4XEI5|C4XEI5_MYCFP    MFIKVILVNSMDRMQMKDCTSVGFSAKIQFINKMKGKYYMAKQDFNRNKDHVNIGTIGHV     60
SP|Q74JU6|EFTU_LACJO      --------------------------------------MAEKEHYERTKPHVNIGTIGHV     22
SP|P64031|EFTU_STRR6      ---------------------------------------MAKEKYDRSKPHVNIGTIGHV     21
SP|A8AWA0|EFTU_STRGC      ---------------------------------------MAKEKYDRSKPHVNIGTIGHV     21
TR|V7KDM5|V7KDM5_MYCPC    ---------------------------------------MAKAKFERTKPHVNIGTIGHV     21
SP|Q6GBT9|EFTU_STAAS      ---------------------------------------MAKEKFDRSKEHANIGTIGHV     21
SP|Q5HRK4|EFTU_STAEQ      ---------------------------------------MAKEKFDRSKEHANIGTIGHV     21
SP|B7IT17|EFTU_BACC2      ---------------------------------------MAKAKFERSKPHVNIGTIGHV     21
SP|P33166|EFTU_BACSU      ---------------------------------------MAKEKFDRSKSHANIGTIGHV     21
SP|Q8Y422|EFTU_LISMO      ---------------------------------------MAKEKFDRSKPHVNIGTIGHV     21
SP|P56003|EFTU_HELPY      ---------------------------------------MAKEKFNRTKPHVNIGTIGHV     21
SP|Q2A1M0|EFTU_FRATH      ---------------------------------------MAKEKFERSKPHVNVGTIGHV     21
TR|X5HZZ4|X5HZZ4_9BURK    ---------------------------------------MAKGKFERTKPHVNVGTIGHV     21
TR|N9JNN0|N9JNN0_ACIB2    ---------------------------------------MAKAKFERNKPHVNVGTIGHV     21
SP|P09591|EFTU_PSEAE      ---------------------------------------MAKEKFERNKPHVNVGTIGHV     21
SP|A6TEX7|EFTU_KLEP7      ---------------------------------------MSKEKFERTKPHVNVGTIGHV     21

SP|P23568|EFTU_MYCPN      DHGKTTLTAAICTVLAKE---GKSAATRYDQIDKAPEEKARGITINSAHVEYSSDKRHYA     78
TR|C4XEI5|C4XEI5_MYCFP    DHGKTTLTAAIATVLSKK---GLAEAKDYAAIDNAPEEKARGITINTSHIEYETEKRHYA     117
SP|Q74JU6|EFTU_LACJO      DHGKTTLTAAITTVLAED---GLAQAEDYSQIDAAPEEKERGITINTAHVEYETKNRHYA     79
SP|P64031|EFTU_STRR6      DHGKTTLTAAITTVLARRLPSSVNQPKDYASIDAAPEERERGITINTAHVEYETEKRHYA     81
SP|A8AWA0|EFTU_STRGC      DHGKTTLTAAITTVLARRLPSSVNQPKDYASIDAAPEERERGITINTAHVEYETEKRHYA     81
TR|V7KDM5|V7KDM5_MYCPC    DHGKTTLTAAITKVLHDKYPD-LNESRAFDQIDNAPEERQRGITINISHVEYQTDKRHYA     80
SP|Q6GBT9|EFTU_STAAS      DHGKTTLTAAIATVLAKN--G-DSVAQSYDMIDNAPEEKERGITINTSHIEYQTDKRHYA     78
SP|Q5HRK4|EFTU_STAEQ      DHGKTTLTAAIATVLAKN--G-DTVAQSYDMIDNAPEEKERGITINTAHIEYQTDKRHYA     78
SP|B7IT17|EFTU_BACC2      DHGKTTLTAAITTVLAKA--G-GAEARGYDQIDAAPEERERGITISTAHVEYETETRHYA     78
SP|P33166|EFTU_BACSU      DHGKTTLTAAITTVLHKKS-G-KGTAMAYDQIDGAPEERERGITISTAHVEYETETRHYA     79
```

```
SP|Q8Y422|EFTU_LISMO      DHGKTTLTAAITTVLAKK--G-YADAQAYDQIDGAPEERERGITISTAHVEYQTDSRHYA   78
SP|P56003|EFTU_HELPY      DHGKTTLSAAISAVLSLK--G-LAEMKDYDNIDNAPEEKERGITIATSHIEYETENRHYA   78
SP|Q2A1M0|EFTU_FRATH      DHGKTTLTAAITKVMAEK--N-GGMARKFDEIDSAPEEKARGITINTSHVEYESPNRHYA   78
TR|X5HZZ4|X5HZZ4_9BURK    DHGKTTLTAAIATVLSAK--F-GGEAKKYDEIDAAPEEKARGITINTAHVEYETANRHYA   78
TR|N9JNN0|N9JNN0_ACIB2    DHGKTTLTAAIATICAKT--Y-GGEAKDYSQIDSAPEEKARGITINTSHVEYDSPTRHYA   78
SP|P09591|EFTU_PSEAE      DHGKTTLTAALTKVCSDT--W-GGSARAFDQIDNAPEEKARGITINTSHVEYDSAVRHYA   78
SP|A6TEX7|EFTU_KLEP7      DHGKTTLTAAITTVLAKT--Y-GGSARAFDQIDNAPEEKARGITINTSHVEYDTPTRHYA   78
                          *******:**:  :              :  ** ****: *****  :*:**.:  ****

SP|P23568|EFTU_MYCPN      HVDCPGHADYIKNMITGAAQMDGAILVVSATDSVMPQTREHILLARQVGVPRMVVFLNKC   138
TR|C4XEI5|C4XEI5_MYCFP    HVDCPGHADYIKNMITGAAQMDGAILVVAATDGAMPQTREHILLSKQVGVPRMVVFLNKC   177
SP|Q74JU6|EFTU_LACJO      HMDAPGHADYIKNMITGAAQMDGAILVVAATDGPMPQTREHILLARQVGVQYIVVFLNKV   139
SP|P64031|EFTU_STRR6      HIDAPGHADYVKNMITGAAQMDGAILVVASTDGPMPQTREHILLSRQVGVKHLIVFMNKV   141
SP|A8AWA0|EFTU_STRGC      HIDAPGHADYVKNMITGAAQMDGAILVVASTDGPMPQTREHILLSRQVGVKHLIVFMNKV   141
TR|V7KDM5|V7KDM5_MYCPC    HVDAPGHADYIKNMITGAAQMDGAILVVAATDGPMPQTREHVLLARQVGVPYILVALNKA   140
SP|Q6GBT9|EFTU_STAAS      HVDCPGHADYVKNMITGAAQMDGGILVVSAADGPMPQTREHILLSRNVGVPALVVFLNKV   138
SP|Q5HRK4|EFTU_STAEQ      HVDCPGHADYVKNMITGAAQMDGGILVVSAADGPMPQTREHILLSRNVGVPALVVFLNKV   138
SP|B7IT17|EFTU_BACC2      HVDCPGHADYVKNMITGAAQMDGGILVVSAADGPMPQTREHILLSRQVGVPYIVVFLNKC   138
SP|P33166|EFTU_BACSU      HVDCPGHADYVKNMITGAAQMDGAILVVSAADGPMPQTREHILLSKNVGVPYIVVFLNKC   139
SP|Q8Y422|EFTU_LISMO      HVDCPGHADYVKNMITGAAQMDGAILVVSAADGPMPQTREHILLSRQVGVPYIVVFMNKC   138
SP|P56003|EFTU_HELPY      HVDCPGHADYVKNMITGAAQMDGAILVVSAADGPMPQTREHILLSRQVGVPHIVVFLNKQ   138
SP|Q2A1M0|EFTU_FRATH      HVDCPGHADYVKNMITGAAQMDGAILVCSAADGPMPQTREHILLSRQVGVPKIVVFLNKC   138
TR|X5HZZ4|X5HZZ4_9BURK    HVDCPGHADYVKNMITGAAQMDGAILVCSAADGPMPQTREHILLARQVGVPYIIVFLNKC   138
TR|N9JNN0|N9JNN0_ACIB2    HVDCPGHADYVKNMITGAAQMDGAILVCAATDGPMPQTREHILLSRQVGVPYIIVFLNKC   138
SP|P09591|EFTU_PSEAE      HVDCPGHADYVKNMITGAAQMDGAILVCSAADGPMPQTREHILLSRQVGVPYIVVFLNKA   138
SP|A6TEX7|EFTU_KLEP7      HVDCPGHADYVKNMITGAAQMDGAILVVAATDGPMPQTREHILLGRQVGVPYIIVFLNKC   138
                          *:*.******:************.*** :::*. *******:**.::***  ::* :** 

SP|P23568|EFTU_MYCPN      DIATD-EEVQELVAEEVRDLLTSYGFDGKNTPIIYGSALKALEGDPK-----WEAKIHDL   192
TR|C4XEI5|C4XEI5_MYCFP    DMLKGEEEMIELVEMEVRELLSKYGFDGDNTPVIRGSALEALKGNKE-----YEDKIMEL   232
SP|Q74JU6|EFTU_LACJO      DLVDD-PELIDLVEMEVRDLLSEYDYPGDDVPVIRGSALKALEGDPE-----QQDVIRKL   193
```

```
SP|P64031|EFTU_STRR6      DLVDD-EELLELVEMEIRDLLSEYDFPGDDLPVIQGSALKALEGDSK-----YEDIVMEL   195

SP|A8AWA0|EFTU_STRGC      DLVDD-EELLELVEMEIRDLLSEYDFPGDDLPVIQGSALKALEGDSK-----YEDIIMDL   195

TR|V7KDM5|V7KDM5_MYCPC    DMVDD-EELLELVEMEVRELLAAQEFD-EDAPVVRVSALKALEGDAK-----WVESVEQL   193

SP|Q6GBT9|EFTU_STAAS      DMVDD-EELLELVEMEVRDLLSEYDFPGDDVPVIAGSALKALEGDAQ-----YEEKILEL   192

SP|Q5HRK4|EFTU_STAEQ      DMVDD-EELLELVEMEVRDLLSEYDFPGDDVPVIAGSALKALEGDAE-----YEQKILDL   192

SP|B7IT17|EFTU_BACC2      DMVDD-EELLELVEMEVRDLLSEYGFPGDDIPVIKGSALKALQGEAD-----WEAKIIEL   192

SP|P33166|EFTU_BACSU      DMVDD-EELLELVEMEVRDLLSEYDFPGDDVPVVKGSALKALEGDAE-----WEAKIFEL   193

SP|Q8Y422|EFTU_LISMO      DMVDD-EELLELVEMEIRDLLTEYEFPGDDIPVIKGSALKALQGEAD-----WEAKIDEL   192

SP|P56003|EFTU_HELPY      DMVDD-QELLELVEMEVRELLSAYEFPGDDTPIVAGSALRALEEAKAGNVGEWGEKVLKL   197

SP|Q2A1M0|EFTU_FRATH      DMVDD-EELLELVEMEVRELLDQYEFPGDDTPVIMGSALRAIEGDEAY-----VEKIVEL   192

TR|X5HZZ4|X5HZZ4_9BURK    DMVDD-EELLELVEMEVRELLDKYDFPGDDTPIVRGSAKLALEGDKG-PLG--EQAIDKL   194

TR|N9JNN0|N9JNN0_ACIB2    DLVDD-EELLELVEMEVRELLSTYDFPGDDTPVIRGSALAALNGEAG-PYG--EESVLAL   194

SP|P09591|EFTU_PSEAE      DMVDD-AELLELVEMEVRDLLNTYDFPGDDTPIIIGSALMALEGKDDNGIG--VSAVQKL   195

SP|A6TEX7|EFTU_KLEP7      DMVDD-EELLELVEMEVRELLSQYDFPGDDTPIVRGSALKALEGDAEWEA-----KIIEL   192
                          *:  .  *: :**  *:*:**    :  .: *::  **  *::            :  *

SP|P23568|EFTU_MYCPN      MNAVDEWIPTPEREVDKPFLLAIEDTMTITGRGTVVTGRVERGELKVGQEIEIVGLRPIR   252

TR|C4XEI5|C4XEI5_MYCFP    MNAVDTWIQTPVKEFDKPFLMAVEDVFTITGRGTVATGRVERGRLNLNEEVEIVGLHPTK   292

SP|Q74JU6|EFTU_LACJO      METVDEYIPTPERDTDKPFLMPVEDVFTITGRGTVASGRIDRGTVKVGDEVEIVGLTDKI   253

SP|P64031|EFTU_STRR6      MNTVDEYIPEPERDTDKPLLLPVEDVFSITGRGTVASGRIDRGIVKVNDEIEIVGIKEET   255

SP|A8AWA0|EFTU_STRGC      MNTVDEYIPEPERDTDKPLLLPVEDVFSITGRGTVASGRIDRGIVKVNDEIEIVGIKEEI   255

TR|V7KDM5|V7KDM5_MYCPC    MEAVDESIPDPVRETDKPFLMPVEDVFTITGRGTVVTGRVERGVINVNEEVEIVGIRPSS   253

SP|Q6GBT9|EFTU_STAAS      MEAVDTYIPTPERDSDKPFMMPVEDVFSITGRGTVATGRVERGQIKVGEEVEIIGLHDTS   252

SP|Q5HRK4|EFTU_STAEQ      MQAVDDYIPTPERDSDKPFMMPVEDVFSITGRGTVATGRVERGQIKVGEEVEIIGMHETS   252

SP|B7IT17|EFTU_BACC2      MTEVDAYIPTPERETDKPFLMPIEDVFSITGRGTVATGRVERGIVKVGDVVEIIGLAEEN   252

SP|P33166|EFTU_BACSU      MDAVDEYIPTPERDTEKPFMMPVEDVFSITGRGTVATGRVERGQVKVGDEVEIIGLQEEN   253

SP|Q8Y422|EFTU_LISMO      MEAVDSYIPTPERDTDKPFMMPVEDVFSITGRGTVATGRVERGQVKVGDEVEVIGIEEES   252

SP|P56003|EFTU_HELPY      MAEVDAYIPTPERDTEKTFLMPVEDVFSIAGRGTVVTGRIERGVVKVGDEVEIVGIRPTQ   257

SP|Q2A1M0|EFTU_FRATH      VQAMDDYIPAPERDTEKPFILPIEDVFSISGRGTVVTGRIERGVVNVGDEVEVVGIRPTQ   252

TR|X5HZZ4|X5HZZ4_9BURK    AEALDTYIPTPERAVDGAFLMPVEDVFSISGRGTVVTGRVERGIIKVGEEIEIVGIRDTQ   254

TR|N9JNN0|N9JNN0_ACIB2    VAALDSYIPEPERAIDKAFLMPIEDVFSISGRGTVVTGRVEAGIIKVGEEVEIVGIKDTV   254
```

```
SP|P09591|EFTU_PSEAE        VETLDSYIPEPVRAIDQPFLMPIEDVFSISGRGTVVTGRVERGIIKVQEEVEIVGIKATT   255
SP|A6TEX7|EFTU_KLEP7        AGHLDTYIPEPERAIDKPFLLPIEDVFSISGRGTVVTGRVERGIIKVGEEVEIVGIKETA   252
                               :*   *  * :  :  ::: :**.::*:*****.:**:: * ::: : :*::*:

SP|P23568|EFTU_MYCPN        -KAVVTGIEMFKKELDSAMAGDNAGVLLRGVDRKEVERGQVLAKPGSIKPHKKFKAEIYA   311
TR|C4XEI5|C4XEI5_MYCFP      -KTVVTGMEMFRKNLKEVQAGDNAGLLLRGIERAGIERGQVLAKPGTIIPHTEFTAAIYV   351
SP|Q74JU6|EFTU_LACJO        EKSTVTGLEMFHKTLDLGEAGDNVGVLLRGIDRDQVERGQVLAAPGSIQTHKNFKGQVYI   313
SP|P64031|EFTU_STRR6        QKAVVTGVEMFRKQLDEGLAGDNVGVLLRGVQRDEIERGQVIAKPGSINPHTKFKGEVYI   315
SP|A8AWA0|EFTU_STRGC        QKAVVTGVEMFRKQLDEGLAGDNVGVLLRGIQRDEIERGQVIAKPGSINPHTKFKGEVYI   315
TR|V7KDM5|V7KDM5_MYCPC      TKTTVTGVEMFRKLLDQGQAGDNVGLLLRGIKREDVERGQVVTKPGTTTPHTEFEGQVYI   313
SP|Q6GBT9|EFTU_STAAS        -KTTVTGVEMFRKLLDYAEAGDNIGALLRGVAREDVQRGQVLAAPGSITPHTEFKAEVYV   311
SP|Q5HRK4|EFTU_STAEQ        -KTTVTGVEMFRKLLDYAEAGDNIGALLRGVAREDVQRGQVLAAPGSITPHTKFKAEVYV   311
SP|B7IT17|EFTU_BACC2        ASTTVTGVEMFRKLLDQAQAGDNIGALLRGVAREDIQRGQVLAKTGSVKAHAKFKAEVFV   312
SP|P33166|EFTU_BACSU        KKTTVTGVEMFRKLLDYAEAGDNIGALLRGVSREEIQRGQVLAKPGTITPHSKFKAEVYV   313
SP|Q8Y422|EFTU_LISMO        KKVVVTGVEMFRKLLDYAEAGDNIGALLRGVAREDIQRGQVLAKPGSITPHTNFKAETYV   312
SP|P56003|EFTU_HELPY        -KTTVTGVEMFRKELEKGEAGDNVGVLLRGTKKEEVERGMVLCKPGSITPHKKFEGEIYV   316
SP|Q2A1M0|EFTU_FRATH        -KTTVTGVEMFRKLLDRGEAGDNVGILVRGLKRDDVERGQVLCKPGSIKPHTKFEAEVYV   311
TR|X5HZZ4|X5HZZ4_9BURK      -KTTCTGVEMFRKLLDQGQAGDNVGLLLRGTKREDVERGQVLCKPGSIKPHTHFTAEVYV   313
TR|N9JNN0|N9JNN0_ACIB2      -KTTVTGVEMFRKLLDEGRAGENCGILLRGTKREEVQRGQVLAKPGTIKPHTKFDAEVYV   313
SP|P09591|EFTU_PSEAE        -KTTCTGVEMFRKLLDEGRAGENVGILLRGTKREDVERGQVLAKPGTIKPHTKFECEVYV   314
SP|A6TEX7|EFTU_KLEP7        -KTTCTGVEMFRKLLDEGRAGENVGVLLRGIKREEIERGQVLAKPGTINPHTKFESEVYI   311
                             . . **:***:* *.   **:* * *:**  :  ::** *:   *:   * .*    :

SP|P23568|EFTU_MYCPN        LKKEEGGRHTGFLNGYRPQFYFRTTDVTGSISLPENTEMVLPGDNTSITVELIAPIACEK   371
TR|C4XEI5|C4XEI5_MYCFP      LTKDEGGRHTPFFKNYKPQFYFRTTDVTGGVEFEKGREMVTPGENVNLTVKLISPIAVEN   411
SP|Q74JU6|EFTU_LACJO        LNKDEGGRHTPFFSDYRPQFYHTTDVTGKIELPEGTEMVMPGDNVEFTVELIKPVAIEK   373
SP|P64031|EFTU_STRR6        LTKEEGGRHTPFFNNYRPQFYFRTTDVTGSIELPAGTEMVMPGDNVTIDVELIHPIAVEQ   375
SP|A8AWA0|EFTU_STRGC        LTKEEGGRHTPFFNNYRPQFYFRTTDVTGSIELPAGTEMVMPGDNVTIEVELIHPIAVEQ   375
TR|V7KDM5|V7KDM5_MYCPC      LSKDEGGRHTPFFNNYRPQFYFRTTDVTGVVTLPEGTEMVMPGDNTNISVKLIQPVAMDE   373
SP|Q6GBT9|EFTU_STAAS        LSKDEGGRHTPFFSNYRPQFYFRTTDVTGVVHLPEGTEMVMPGDNVEMTVELIAPIAIED   371
SP|Q5HRK4|EFTU_STAEQ        LSKDEGGRHTPFFTNYRPQFYFRTTDVTGVVNLPEGTEMVMPGDNVEMTVELIAPIAIED   371
```

```
SP|B7IT17|EFTU_BACC2      LSKEEGGRHTPFFANYRPQFYFRTTDVTGIIQLPEGTEMVMPGDNIEMTIELIAPIAIEE   372
SP|P33166|EFTU_BACSU      LSKEEGGRHTPFFSNYRPQFYFRTTDVTGIIHLPEGVEMVMPGDNTEMNVELISTIAIEE   373
SP|Q8Y422|EFTU_LISMO      LTKEEGGRHTPFFNNYRPQFYFRTTDVTGIVTLPEGTEMVMPGDNIELAVELIAPIAIED   372
SP|P56003|EFTU_HELPY      LSKEEGGRHTPFFTNYRPQFYVRTTDVTGSITLPEGVEMVMPGDNVKITVELISPVALEL   376
SP|Q2A1M0|EFTU_FRATH      LSKEEGGRHTPFFKGYRPQFYFRTTDITGAVELPEGVEMVMPGDNVKMTITLINPIAMDE   371
TR|X5HZZ4|X5HZZ4_9BURK    LSKDEGGRHTPFFNNYRPQFYFRTTDVTGAIELPADKEMVMPGDNVSITVKLINPIAMEE   373
TR|N9JNN0|N9JNN0_ACIB2    LSKEEGGRHTPFLNGYRPQFYFRTTDVTGAIQLKEGVEMVMPGDNVEMSVELIHPIAMDP   373
SP|P09591|EFTU_PSEAE      LSKEEGGRHTPFFKGYRPQFYFRTTDVTGNCELPEGVEMVMPGDNIKMVVTLIAPIAMED   374
SP|A6TEX7|EFTU_KLEP7      LSKDEGGRHTPFFKGYRPQFYFRTTDVTGTIELPEGVEMVMPGDNIKMVVTLIHPIAMDD   371
                          *.*:****** *: .*:****.:***:**   :  . *** **:*  : : **  :* :

SP|P23568|EFTU_MYCPN      GSKFSIREGGRTVGAGSVTEVLE   394
TR|C4XEI5|C4XEI5_MYCFP    GTKFSIREGGRTVGYGNVTKIIK   434
SP|Q74JU6|EFTU_LACJO      GTKFTIREGGKTVGAGQVTEILD   396
SP|P64031|EFTU_STRR6      GTTFSIREGGRTVGSGMVTEIEA   398
SP|A8AWA0|EFTU_STRGC      GTTFSIREGGRTVGSGMVTEIEA   398
TR|V7KDM5|V7KDM5_MYCPC    GLRFAIREGGRTVGAGRVVKIIK   396
SP|Q6GBT9|EFTU_STAAS      GTRFSIREGGRTVGSGVVTEIIK   394
SP|Q5HRK4|EFTU_STAEQ      GTRFSIREGGRTVGSGVVTEIFE   394
SP|B7IT17|EFTU_BACC2      GTKFSIREGGRTVGYGVVATIVE   395
SP|P33166|EFTU_BACSU      GTRFSIREGGRTVGSGVVSTITE   396
SP|Q8Y422|EFTU_LISMO      GTKFSIREGGRTVGAGVVSNISK   395
SP|P56003|EFTU_HELPY      GTKFAIREGGRTVGAGVVSNIIE   399
SP|Q2A1M0|EFTU_FRATH      GLRFAIREGGRTVGAGVVAKIIE   394
TR|X5HZZ4|X5HZZ4_9BURK    GLRFAIREGGRTVGAGVVAKIIA   396
TR|N9JNN0|N9JNN0_ACIB2    GLRFAIREGGRTVGAGVVAKVTA   396
SP|P09591|EFTU_PSEAE      GLRFAIREGGRTVGAGVVAKIIE   397
SP|A6TEX7|EFTU_KLEP7      GLRFAIREGGRTVGAGVVAKVLG   394
                          *  *:*****:*** * *  :
```
